# Ferrocene-Containing Conjugated Oligomers Synthesized by Acyclic Diene Metathesis Polymerization

**DOI:** 10.3390/polym11081334

**Published:** 2019-08-12

**Authors:** Xin Gao, Lei Deng, Jianfeng Hu, Hao Zhang

**Affiliations:** 1 College of Chemistry & Chemical Engineering, Inner Mongolia University, Hohhot 010021, China; 2 Inner Mongolia Key Laboratory of Fine Organic Synthesis, Hohhot 010021, China

**Keywords:** ferrocene, conjugated oligomers, acyclic diene metathesis (ADMET) polymerization

## Abstract

A series of conjugated, symmetrical, and ferrocene-containing main-chain monomers was prepared following a gentle coupling reaction. Ferrocene-containing oligomers with all-*trans*-configured vinylene bonds could be synthesized via acyclic diene metathesis (ADMET) polymerization. These oligomers had a larger Stokes shift (2400 to 2600 cm^−1^) and both exhibited stable and reversible electrochemistry. Meanwhile, the copolymerization of 1,1’-bis[1-methyl-2-(4-vinylphenyl)ethenyl]ferrocene with 2,7-divinyl-9,9-dioctylfluorene was achieved. The structurally regular copolymers proved their optical and electrochemical properties. The fluorescence intensity of the copolymer gradually enhanced with the increasing number of fluorene units. At the same time, it was also found that the color of the copolymers had a significant change from yellow-green to red.

## 1. Introduction

**Scheme 1:**
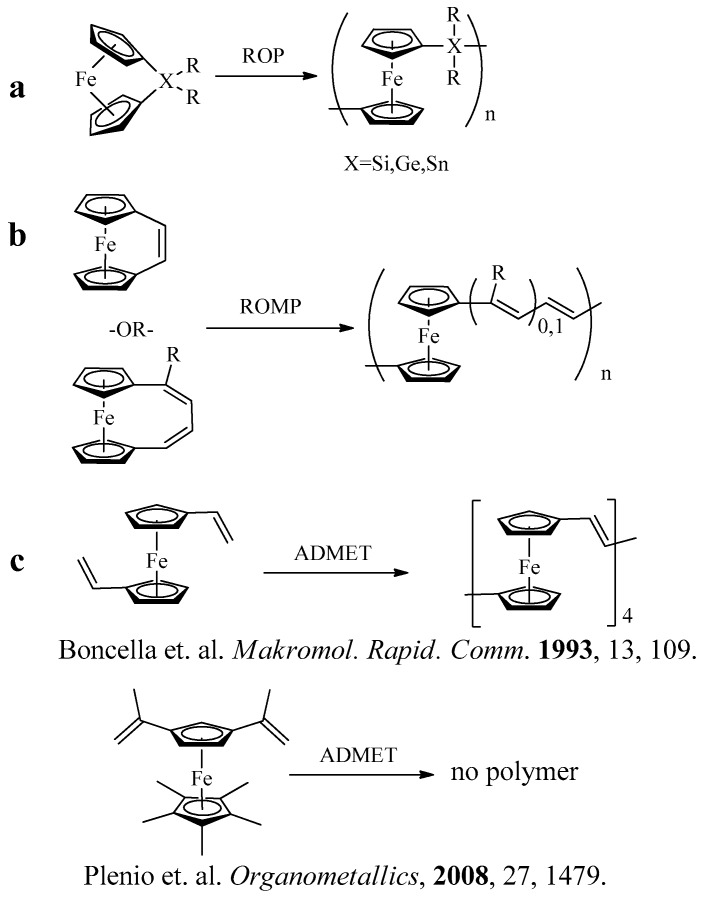
General polymerization method for synthesizing polyferrocenes.

Ferrocene is the most stable metallocene with a steady 18-electron structure and the highest average dissociation energy, which is the trait of being impervious to air and humidity [1]. Therefore, since the first example of polyferrocenes was reported in 1955, the study of ferrocene-containing polymers has intrigued chemists and blossomed into a mature field [2,3,4,5,6,7,8,9,10,11,12,13,14,15,16,17,18]. These polymers are sought due to their useful properties, which range from catalysis to magnetism and electrochemical characteristics [19,20,21]. Generally, there are several methods for transitioning metal-containing polymers in the main chain, such as ring-opening [4,18,22,23,24] polymerization (ROP) (Scheme 1a), ring opening metathesis [25,26,27,28,29] polymerization (ROMP) (Scheme 1b), and acyclic diene metathesis [30,31,32] (ADMET) polymerization (Scheme 1c). Up to now, only a few papers about ADMET polymerization have been reported, comparing ROP and ROMP. Their results were unsatisfactory because of low molecular weight or negative experimental results.

Over the past decade, transition metal-containing polymers, especially oligomers, have attracted much interest due to their applications. On first reflection, the incorporation of metals into polymers naturally enhanced conductivity, given the high values associated with the metallic state [7,16]. Chen et al. [33] and Swager group [34] prepared polymers successfully showing conductivity. Metallated conjugated polymers have also demonstrated exceptional promise in the creation of high-efficiency polymer solar cells [16]. Recently, great efforts have been dedicated to developing new oligomer molecules for applications in solar cells as active layer materials, including electron donors and electron acceptors [35].

**Scheme 2:**
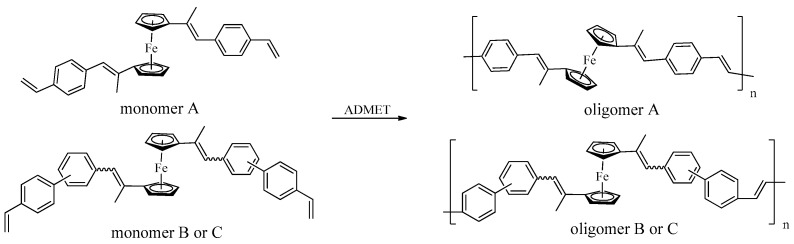
Ferrocene-containing conjugated oligomers synthesized by acyclic diene metathesis (ADMET) polymerization.

Conjugate polymers are promising macromolecular compounds, owing to their potential applications in electrochemical devices and optical properties [36,37,38,39]. However, conjugate monomers are rarely used in ADMET polymerization. Therefore, the discovery of new conjugate compounds containing ferrocene as monomers in ADMET polymerization is also desirable. In this paper, the successful synthesis of a series of conjugated, symmetrical, and ferrocene-containing monomers using a metal-free carbon–carbon bond-forming coupling reaction was reported [40]. Using ADMET strategy, the polymerization (Scheme 2) of a series of ferrocene-containing conjugated monomers and copolymerization with 2,7-divinyl-9,9-dioctylfluorene (monomer D) were carried out. The properties of oligomers and copolymers were characterized.

## 2. Materials and Methods 

All hydrocarbon solvents were distilled from sodium before use. All reagents were used as received from commercial sources, unless otherwise noted, and the Grubbs type catalysts were prepared according to the literature [41,42]. All experiments were carried out under a nitrogen atmosphere in a dry box and conventional Schlenk line techniques unless otherwise specified. All ^1^H and ^13^C NMR spectra were recorded on a Bruker 500 spectrometer (499.65 MHz, 1H). The polymer samples for analysis were prepared by dissolving the polymers in CDCl_3_ solution and the spectra were measured at 25 °C. Number- (M_n_) and weight- (M_w_) averaged molecular weights and polydispersity indices (M_w_/M_n_) of the polymers were estimated by a Waters 2545 instrument equipped with four Waters Styragel HR columns, i.e., HR-1, HR-3, HR-4, and HR-5E. HPLC grade THF was used as eluent at a flow rate of 1.0 mL/min at 35 °C. IR spectra were recorded on a Nicolet nexus 670 FT-IR spectrophotometer. UV spectra were measured on an FLS 920 spectrophotometer. Fluorescence spectra were recorded with a HitachiF-7000 spectrofluorimeter. Thermogravimetric analysis (TGA) measurements were carried out under nitrogen on a Perkin-Elmer TGA 7 analyzer at a heating rate of 20 °C ·min^−1^. DSC measurements were performed on a Mettler-Toledo S1. Cyclic voltammetry (CV) analyses were recorded on an autolab–pgstat (model 302). The samples for electron microscopy were prepared by drop casting 1 drop of suspension of the sample onto a carbon coated copper grid, which was placed on a piece of filter paper to remove excess solvent. Bright field transmission electron microscopy (TEM) micrographs were obtained both on a JEOL1200EX TEM Mk1 and Mk2 microscope operating at 120 kV.

### 2.1. Synthesis of 1,1’-Diacetylferrocene (2)

The AlCl_3_ (8.97 g, 0.07 mol) was added to 1,2-dichloroethane (30 mL) and stirred at room temperature. The solution of acetylchloride (6.33 g, 0.08 mol) in 1,2–dichloroethane (8.4 mL) was dropwise added into the above solution and stirred at 0 °C in a three-necked flask. The solution of ferrocene (5 g, 0.03 mol) in 1,2-dichloroethane (40 mL) was added to the flask to give the mixture as a purple complex and stirred at 0 °C for 3 h. Then the solution was restored to room temperature and stirred at 40 °C for 1 h. The mixture was poured into ice water and diluted with 1,2-dichloroethane. The organic layer was dried over MgSO_4_ and evaporated to dryness under reduced pressure. The residue was purified by column chromatography (PE: EA = 10:1) to give 2 as a red solid (4.52 g, 62% yield).

### 2.2. Synthesis of N’,N’’-(1,1’-ferrocenylbis(ethan-1-yl-1-ylidene))bis(4-methylbenzenesulfonohydrazide) (3)

The solution of 2 (3.52 g, 13 mmol) in methanol (16 mL) was dropwise added to the solution of methylbenzenesulfonhydrazide (10.55 g, 56 mmol) in methanol (56 mL) at 60 °C for 4 h. Then the mixture was washed with PE to give 3 as a yellow solid (7.56 g, 95% yield).

### 2.3. Synthesis of Monomer A 

In a glovebox, a toluene solution (5 mL) of Pd_2_(dba)_3_ (9.2 mg, 5.0 mol%), P-(4-MePh)_3_ (6.1 mg, 10 mol%), 3 (121.2 mg, 0.20 mmol), and LiO*^t^*Bu (96.1 mg, 6 equiv) were added into a 25 mL Schlenk tube, then 4-vinylbenzyl chloride was added to the mixture. The Schlenk tube was sealed and taken out of the glovebox and then heated at 70 °C for 12 h. Then, the solution was evaporated to dryness under reduced pressure and the mixture was purified by column chromatography (PE as eluent) to give monomer A as a red solid (48.2 mg, 41% yield, Z: E = 1:3). ^1^H NMR (500 MHz, CDCl_3_) δ 7.33 (d, *J* = 8.0 Hz, 3H), 7.20 (d, *J* = 8.1 Hz, 3H), 6.74–6.70 (m, 2H), 6.68 (s, 2H), 5.75 (d, *J* = 17.7 Hz, 2H), 5.24 (d, *J* = 10.9 Hz, 2H), 4.46 (s, 4H), 4.27 (s, 4H), 2.19 (s, 6H). ^13^C NMR (126 MHz, CDCl_3_), δ 138.0, 136.7, 135.3, 134.8, 129.2, 128.2, 126.1, 123.4, 113.4, 90.1, 70.0, 66.9, 17.3.

### 2.4. Synthesis of Monomer B 

In a glovebox, a solution of Pd_2_(dba)_3_ (9.2 mg, 5.0 mol%), PCy_3_ (5.6 mg, 10 mol%), 3 (121.2 mg, 0.20 mmol), and LiO*^t^*Bu (96.1 mg, 6 equiv.) in toluene (3 mL) and dioxane (2 mL) were added into a 25 mL Schlenk tube and stirred at 90 °C for 0.5 h. Then, 4-vinylphenylboronic acid was added to the tube and stirred at 90 °C for 12 h. The mixture was evaporated to dryness under reduced pressure and washed with dichloromethane. The organic layer was dried over MgSO_4_ and evaporated to dryness under reduced pressure. The residue was purified by column chromatography (PE as eluent) to give monomer B as a red solid (74.7 mg, 60% yield, Z: E = 1:1). ^1^H NMR (500 MHz, CDCl_3_) δ 7.65–7.55 (m, 6H), 7.52–7.44 (m, 6H), 7.44–7.39 (m, 6H), 7.37 (d, *J* = 7.9 Hz, 4H), 7.33 (d, *J* = 7.1 Hz, 2H), 7.23 (d, *J* = 8.0 Hz, 4H), 6.72 (s, 2H), 6.46 (s, 2H), 4.42 (s, 4H), 4.26 (s, 4H), 4.16 (s, 4H), 4.13 (s, 4H), 2.29 (s, 6H), 2.21 (s, 6H). ^13^C NMR (126 MHz, CDCl_3_), δ 141.0, 138.7, 138.1, 137.6, 135.0, 134.3, 129.5, 128.9, 128.8, 127.2, 127.0, 126.9, 126.7, 125.9, 123.2, 90.0, 85.7, 70.2, 70.0, 69.6, 67.0, 25.3, 17.3.

### 2.5. Synthesis of Monomer C 

Monomer C was prepared under the same synthetic procedure to that of monomer B. ^1^H NMR (500 MHz, CDCl_3_) δ 7.51 (d, *J* = 8.2 Hz, 4H), 7.46 (d, *J* = 6.4 Hz, 4H), 7.44 (s, 2H), 7.39 (d, *J* = 7.7 Hz, 2H), 7.31 (t, *J* = 7.7 Hz, 2H), 7.23 (d, *J* = 10.1 Hz, 2H), 6.77 (s, 2H), 6.76–6.70 (m, 2H), 5.77 (d, *J* = 17.6 Hz, 2H), 5.26 (d, *J* = 10.9 Hz, 2H), 4.46 (s, 4H), 4.28 (s, 4H), 2.24 (s, 6H). ^13^C NMR (126 MHz, CDCl_3_) δ 140.7, 138.9, 136.6, 136.5, 135.0, 128.6, 128.0, 127.7, 127.3, 126.7, 124.7, 123.5, 113.9, 89.9, 70.1, 67.0, 17.3.

### 2.6. Synthesis of 9,9-dioctyl-2,7-divinylfluorene (Monomer D)

In a glovebox, a solution of 9,9-di-n-octyl-2,7-dibromofluorene (500 mg, 0.91 mmol), tributyl(vinyl)tin (635 mg, 2.0mmol), Pd(PPh_3_)_4_ (42 mg, 3.9 mol%), and a small amount of 2,6-di-tert-butyl-4-methylphenol in toluene (3 mL) was added to a 25 mL Schlenk tube and stirred at 90 °C for 20 h. The solvent was evaporated under reduced pressure and residue was purified by column chromatography (PE as eluent) to give monomer D as a colorless oil (184.6 mg, 48% yield). ^1^H NMR (500 MHz, CDCl_3_) δ 7.63 (d, *J* = 7.8 Hz, 2H), 7.40 (d, *J* = 7.9 Hz, 2H), 7.37 (s, 2H), 6.81 (d, *J* = 10 Hz, 10.9 Hz, 2H), 5.81 (d, *J* = 10 Hz, 2H), 5.26 (d, *J* = 10.9 Hz, 2H), 1.99–1.96 (m, 4H), 1.45–0.91 (m, 20H), 0.84–0.81 (m, 8H).

### 2.7. Synthesis of Oligomer A 

A 100 mL reaction tube was charged with monomer A (0.1 g, 0.21 mmol, 0.21 M) and 1 mL toluene in glovebox. The catalyst solution of Grubbs 2nd catalyst (1.8 mg, 2.1 μmol, 100 μL, 0.02 M) was injected into the reaction tube. The reaction took place under vacuum at 80 °C. After 12 h, the polymerization was quenched by adding a substantial amount of methanol. The reaction mixture was then stirred for 1 h for completion. To end the reaction, 100 mL methanol was added into the reaction solution. The yellow solid oligomer was collected by filtration and was then dried in a vacuum (68 mg, 72% yield). ^1^H NMR (500 MHz, CDCl_3_) δ 7.33–7.29 (d, *J* = 3.8 Hz, 4H), 7.27 (d, *J* = 4.0 Hz, 4H), 7.21 (t, *J* = 7.1 Hz, 2H), 6.73 (s, 2H), 4.46 (s, 4H), 4.28 (s, 4H), 2.21 (s, 6H). ^13^C NMR (126 MHz, CDCl_3_) δ 138.4, 134.6, 129.1, 128.2, 126.0, 123.6, 90.0, 70.0, 66.9, 17.1.

### 2.8. Synthesis of Copolymer 1 

A 100 mL reaction tube was charged with monomer A (0.1 g, 0.21 mmol, 0.21 M), monomer D (0.37 g, 0.84 mmol, 0.84 M), and 1 mL toluene in a glovebox. The catalyst solution of Grubbs second catalyst (1.8 mg, 2.1 μmol, 100 μL, 0.02 M) was injected into the reaction tube. The reaction took place under a vacuum and 60 °C. After 24 h, the polymerization was quenched by adding a substantial amount of methanol. The reaction mixture was then stirred for 1 h for completion. To end the reaction, 100 mL methanol was added into the reaction solution. The sticky yellow copolymer was collected by filtration and was then dried in a vacuum. ^1^H NMR (500 MHz, CDCl_3_) δ 7.68 (d, *J* = 10.0, 4.4 Hz, 2H), 7.54 (t, *J* = 9.9 Hz, 4H), 7.51–7.47 (m, 4H), 7.45 (d, *J* = 6.0, 3.6 Hz, 2H), 7.29 (d, *J* = 9.1 Hz, 4H), 7.22–7.18 (d, *J* = 15.6 Hz, 2H), 6.72 (s, 2H), 4.47 (s, 4H), 4.29 (s, 4H), 2.23 (d, *J* = 24.0 Hz, 6H), 2.04 (s, 5H), 1.65(d, *J* = 15.7, 8.0 Hz, 1H), 1.54 (s, 2H), 1.44 (s, 1H), 1.36 (d, *J* = 7.4 Hz, 3H), 1.26 (s, 5H), 1.19 (s,7H), 1.08 (s, 22H), 0.93 (t, *J* = 7.3 Hz, 2H), 0.81 (s, 10H), 0.69 (s, 5H). ^13^C NMR (126 MHz, CDCl_3_) δ 151.6, 140.7, 138.4, 135.3, 134.9, 134.6, 132.6, 130.6, 129.4, 129.1, 128.2, 127.8, 126.3, 126.0,123.7, 123.4, 120.7, 120.0, 90.0, 70.0, 66.9, 55.1, 31.9, 30.2, 29.8, 29.3, 23.9, 22.7, 17.2, 14.2.

## 3. Results 

### 3.1. Monomer Synthesis and Characterisation

Monomer A, monomer B, and monomer C could be synthesized by the route outlined in Scheme 3. These monomers were fully characterized by ^1^H NMR, ^13^C NMR, and HRMS (Figures S1–S9).

### 3.2. Acyclic Diene Metathesis (ADMET) Polymerization of Monomers

The effect of time, solvent, and type of catalyst on the polymerization was evaluated as shown in Table 1. First of all, the ADMET of monomer A was successfully carried out using the ruthenium-based Grubbs second generation catalyst, oligomer A, with a number-average molecular weight M_n_ = 2001 Da and molecular weight distribution M_w_/M_n_ = 1.10 was obtained (Table 1, Entry 1). The yield of oligomer A increased with the increase in temperature from 80 to 100 °C, but the metathesis of monomer A still only gave oligomers (Table 1, Entry 2). Likewise, further prolonging the reaction time to 24 h resulted in a lower M_n_ = 1614 Da (Table 1, Entry 3). The result (M_n_ = 2021 Da) was similar to Entry 1 using 1,2-dichlorobenzene (DCB) as a solvent with a higher boiling point (Table 1, Entry 4). Finally, we still obtained oligomers with an average of three repeating units using a Grubbs–Hoveyda second generation catalyst and mixed solvents of toluene and DCB (Table 1, Entries 5 to 7). On the basis of monomer A, the optimal conditions of monomers B and C were investigated (Table 1, Entries 8 to 13). Monomers B and C were more rigid than monomer A, so we gained a lower M_n_ of 2160 Da and 3360 Da, respectively. However, above all, the ADMET results with the various monomers all obtained low molecular weight. Low molecular weight may be attributed to limited solubility, which may be caused by ferrocene-containing main-chain oligomers and a lack of alkyl groups. Some related generated ferrocene polymers were reported to be soluble only in the presence of such moieties [27,43,44].

### 3.3. Microstructure via NMR and FT-IR

**Figure 1:**
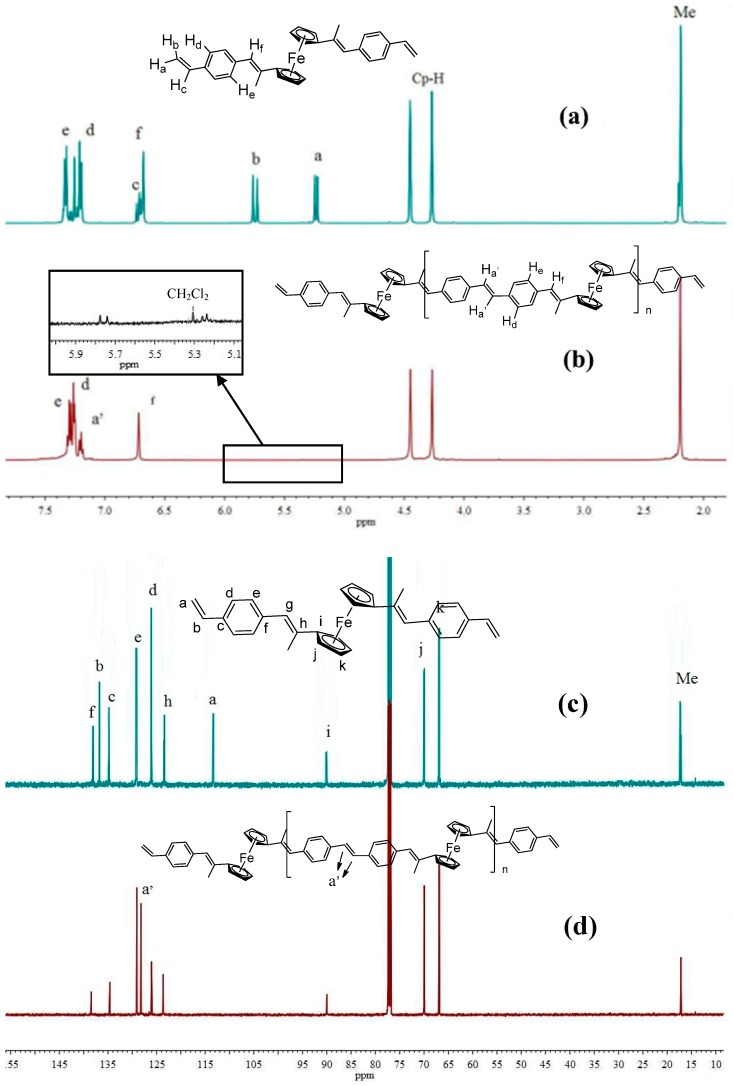
^1^H NMR (500 MHz, CDCl_3_) and ^13^C NMR (125 MHz, CDCl_3_) spectra of (**a**,**c**) monomer A and (**b**,**d**) oligomer A (Table 1, Entry 1).

The microstructure of both the monomers and the oligomers was determined by ^1^H and ^13^C NMR spectra (Figures S20–S25). Respectively, the ^1^H and ^13^C NMR spectra of monomer A and oligomer A were depicted in Figure 1. Their ^1^H NMR spectra were consistent with their structures. The appearance of a resonance at 7.20 ppm manifested the formation of internal *trans*-vinylene protons (–CH_a_’=CH_a_’–) in oligomer A [45,46]. No resonances for a *cis*-configured double bond were observed at 6.5 ppm (reported value for the internal vinylene proton signals in *cis*-stilbene). The aromatic protons appeared at 7.25 and 7.30 ppm. The downfield shift of the aromatic protons in oligomer A relative to monomer A might be due to the extended electron conjugation. It’s worth noting that we could clearly see the terminal vinyl group in the ^1^H NMR spectrum (Figure 1B). Moreover, after ADMET, the ^13^C NMR spectrum of the terminal vinyl group (–C_a_H=C_b_H_2_) at 113.1 and 136.7 ppm disappeared and a new peak emerged at 129.2 ppm, corresponding to the internal vinylene carbons (–C_a_’H=C_a_’H–).

FT-IR spectroscopy showed the obvious differences between monomer A and oligomer A. Figure S26 illustrated this for a select region of the FT-IR spectra of monomer A and its ADMET product. It was obvious that the absorption peaks of C=C stretching at 861 cm^−1^ and 807 cm^−1^ for monomer A disappeared after the ADMET reaction and a new C=C stretching was observed at 989 cm^−1^, strongly indicating that there is a newly generated out-of-plane (*oop*) bending internal *trans*-vinylene bond. Strong evidence from FT-IR, ^1^H, and ^13^C NMR clearly indicated that an exclusively *trans*-configured vinylene bond of stilbene was formed by ADMET polymerization of monomer A.

To further evaluate degree of polymerization, we tested the resulting oligomer by matrix-assisted laser desorption time of flight mass spectrometry (MALDI-TOF MS) (Figure S11). On the basis of this, we could conclude that ions identify with the different molecular weight oligomers with n values between 2 and 5 repetitive individual monomer units, which were in good accordance with the data of GPC.

### 3.4. Optical Property

**Figure 2:**
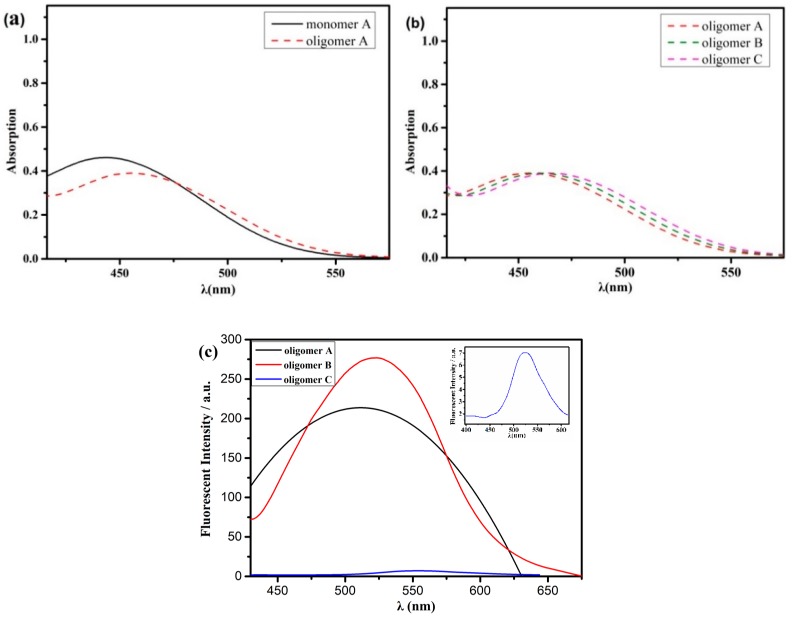
(**a**) UV–Vis spectra of monomer A and oligomer A in CH_2_Cl_2_. (**b**) UV–Vis spectra of oligomers A, B, and C in CH_2_Cl_2_. (**c**) Fluorescence spectra of oligomers A, B, and C in CH_2_Cl_2_ (insert of magnified image of oligomer C).

Oligomer samples with different conjugation repeat units were analyzed with regard to their optical characterization by UV/Vis absorption spectra and fluorescence spectra (Figure 2), recorded as dichloromethane solutions (0.05 mM). There are broad featureless absorption bands of these oligomers in the visible region that could be put down to MLCT transitions, with their absorption maxima between 454 and 468 nm. The emission showed λ_max_ = 510–524 nm for all oligomers, indicating that these oligomers had Stokes shift values ranging from 2400 to 2600 cm^−1^. As expected, the absorption of oligomers was red-shifted compared to the monomers (Figure 2a and Figure S27). On the basis of the above observations, we analyzed the bathochromic shift of the oligomers related to the different chain length and provided evidence for extended conjugation chain lengths. The fluorescence of all oligomers is quenched due to containing ferrocene in the oligomers, as shown in Figure 2c. However, as shown in Figure 2c, on account of the different degrees of polymerization, fluorescence intensity of oligomer C (n = 5) compared to oligomer A and oligomer B (n = 3) dramatically decreased. The difference in fluorescence intensity of oligomer B and oligomer C may be due to the reason that ferrocene is known to act as an efficient quencher [32]. Molar extinction coefficients for new molecules were shown in Table 2. Oligomers A, B, and C had almost extinction coefficient because of the low molecular weight, while the extinction coefficient of copolymer 1 was higher than that of the oligomers.

### 3.5. Thermal Stability Studies

**Figure 3:**
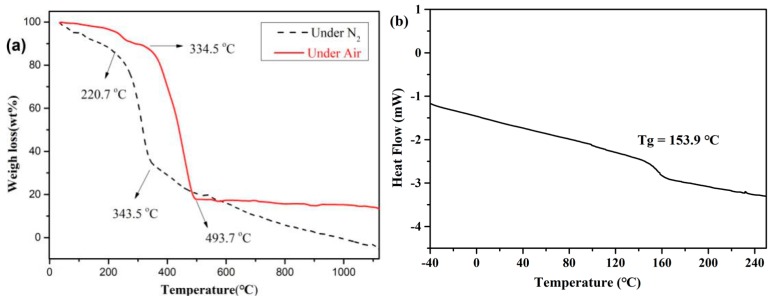
(**a**) Thermogravimetric analysis (TGA) curves of oligomer A under N_2_ and air; (**b**) differential scanning calorimetry (DSC) curve of oligomer A.

In order to test the thermal stability of the oligomers, we then investigated by differential scanning calorimetry (DSC) and thermogravimetric analysis (TGA). Thermogravimetric analysis (Figure 3a) was performed under inert atmosphere (N_2_) and air to compare the stability of oligomer A in a different atmosphere. A similar trend of thermal decomposition behavior was obtained from the oligomer with a different molecular weight. At a scan rate of 10 °C·min^−1^, oligomer A showed good stability up to ca. 334.5 °C under air and it revealed that oligomer A had good resistance to thermolysis. But when we continued to raise the temperature, it led to rapid degrading. Moreover, no appreciable weight loss of oligomer A was found with the TGA analysis up to ≈ 493 °C under air. Oligomer A showed a glass transition temperature (*T_g_*) of 153.9 °C (Figure 3b), whereas the DSC curve showed that oligomer A was amorphous without a clear melting point (*T_m_*).

### 3.6. Electrochemical Properties

**Figure 4:**
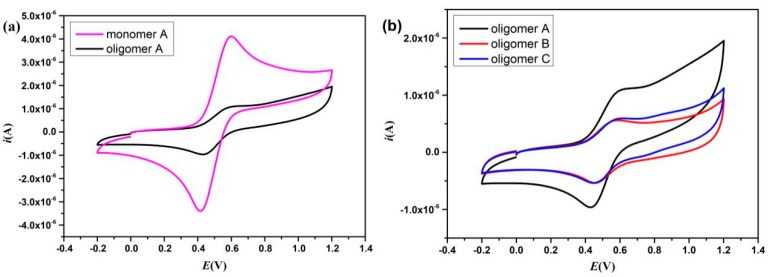
(**a**) Cyclic voltammogram of monomer A and oligomer A. (**b**) Cyclic voltammogram of oligomer A, oligomer B, and oligomer C.

In attempts to see the electrochemical properties of monomers and oligomers, cyclic voltammetry was used (Figure 4 and Figures S36 and S37). The voltammogram was obtained in a CH_2_Cl_2_ solution (0.5 mM) at a scan rate of 100 mV/s, using Bu_4_NPO_4_F_6_ as the supporting electrolyte. As shown in Figure 4, the result demonstrated that stable and reversible electrochemical properties of oligomers could be recorded in an organic solvent. An example of oligomer B, the oxidation process appeared at 0.55 V and the reduction was about 0.48 V, indicating a one-electron transfer reaction (~0.07 V). Similarly, i_pa_/i_pc_ ≈ 1 demonstrated an electrochemical reversibility. The same analysis can be applied to oligomer A and oligomer C (Table 3).

### 3.7. Copolymerization of Monomer A and 9,9-Dioctyl-2,7-Divinylfluorene (Monomer D)

**Scheme 4:**
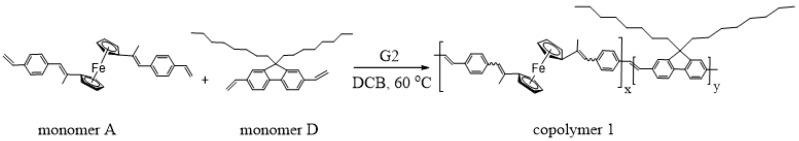
Copolymerization of monomer A with monomer D.

After having proved the validity of the ADMET method, we then examined the copolymerization of monomer A with monomer D (Scheme 4). Some representative results are shown in Table 4. Remarkably, these results of ^1^H NMR and ^13^C NMR spectra suggested that copolymers were obtained successfully (Figures S29 to S32). According to the data of Table 4, it was reasonable to assume that the ratio of incorporation of the ferrocene group could be adjusted by simple variation of the monomer feed ratio.

**Figure 5:**
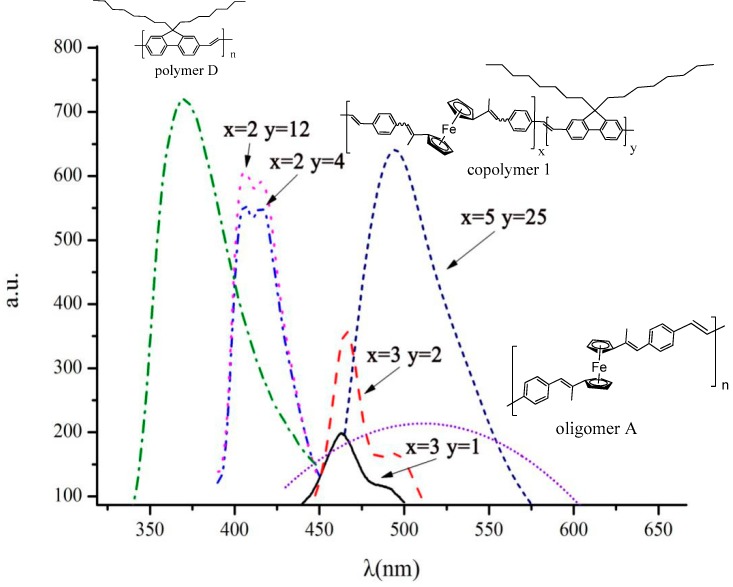
Emission spectra of copolymer 1, oligomer A, and polymer D (5 × 10 ^−5^ M, in CH_2_Cl_2_).

It was interesting to note that fluorescence intensity of copolymers was related to the ratio of fluorene and ferrocene (Figure 5). The fluorescence intensity of copolymer 1 was gradually enhanced with the increasing number of fluorene units. The fluorescence intensity of monomer D was the strongest. The emission spectra of copolymer 1 with different ratios of ferrocene and fluorene all showed two emission bands, which were attributed to the ferrocene unit and fluorene segment, respectively [47]. With the increasing of ferrocene units in copolymer 1, the sharp band gradually widened and passivated. There was only an emission peak when the number of ferrocenes was too high (x = 5, y = 25, Figure 5) in the copolymer because of serious band passivation. Oligomer A without the fluorene segment eventually turned like a parabola. Figure 5 showed the emission of polymer D in the 375 nm, whereas the emission of the copolymer with one or two fluorene units which were assigned to the content of the ferrocene unit was at 460 nm. Owing to increasing content of ferrocene, we could notice that the emission peak became a long wavelength. Meanwhile, it was also found that the color of the copolymer had a significant change from yellow-green to red (Figures S33 and S34) with the monomer D/monomer A feed ratio being 4/1 to 1/4. As shown in Figure S33, we could observe that the state of the copolymer has a gradient process from an oily, semi-solid to a solid with a varying incorporation ratio of monomer D/monomer A. The reason for this phenomenon could be that monomer D was an oil and monomer A was solid. When the relative amount of monomer A in the copolymer was more than that of monomer D, the copolymer would tend to exhibit a solid-like state. Conversely, the copolymer would behave in an oil-like state.

Electrochemical properties are intrinsic properties of ferrocene, so the electrochemical properties of copolymer 1 were probed using cyclic voltammetry (Figure S35). As shown in Table 5, when the number of ferrocenes in polymer was certain, the redox capacity of ferrocene decreased with the increasing number of fluorene units. Therefore, it suggested that the redox ability of copolymers could be adjusted by regulating the ratio of fluorene in the copolymer. This property may be beneficial for the future application of ferrocene-containing polymers.

### 3.8. SEM and TEM Studies

**Figure 6:**
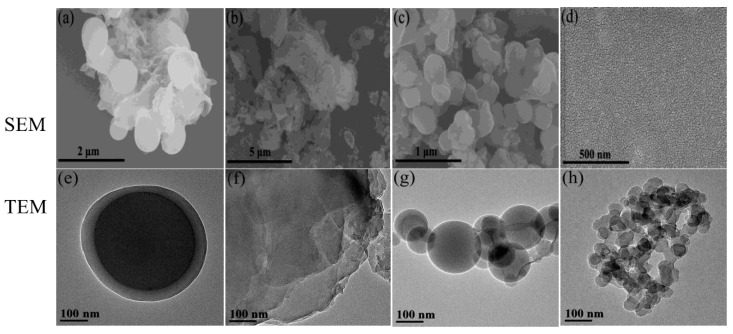
SEM and TEM pictures (**a**,**e**) oligomer A; (**b**,**f**) oligomer B; (**c**,**g**) oligomer C; (**d**,**h**) copolymer 1.

The morphology of oligomers was investigated using SEM and TEM (Figure 6). The morphology of oligomer A basically presented as a regular sphere under SEM and the diameter of the nanosphere was estimated to be about 400 nm from TEM. As shown in Figure 6, because the outer layer of oligomer A was encased in a small molecule oligomer with a lower density, the surface of oligomer A had a 10 nm shell. Oligomer B was layered and oligomer C was a heterogeneous spherical shape with a diameter ranging from 50 to 200 nm. It can be seen in the TEM image of oligomer C that it displayed an orderly arrangement as a chain. The reason for the huge difference in morphology of oligomer B and oligomer C could be the different substitution position of phenyl in monomers. The morphology of the oligomer tending towards a lamellar structure when the phenyl group was substituted in the para-position, corresponding to oligomer B, could be observed in TEM. Copolymer 1 was made up of fine particles with a diameter approaching 50 nm, which was visible in Figure 6. The microstructure and particle size of copolymer 1 after adding monomer D were further optimized in comparison to oligomer A.

## 4. Conclusions

In summary, ferrocene-containing conjugated oligomers were synthesized by ADMET polymerization. The microstructures of oligomers were confirmed by means of ^1^H NMR, ^13^C NMR, FT-IR, and MS. These results showed that the organic conjugated segment had formed with only *trans*-configured vinylene bonds. These oligomers had a larger Stokes shift (2400 to 2600 cm^−1^) and both exhibited stable and reversible electrochemistry in an organic solvent. The oligomers showed good thermal stability, evidenced by TGA and DSC. Moreover, the copolymerization of divinylferrocene (monomer A) and divinylfluorene (monomer D) was successful. Electrochemical properties of the copolymer indicated a negative correlation between the redox capacity of ferrocene and the amount of fluorene.

## Figures and Tables

**Scheme 3:**
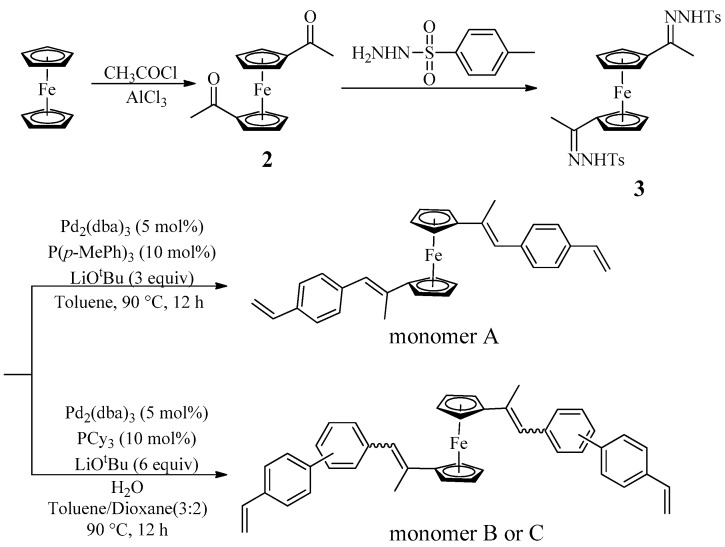
Synthetic route of monomers.

**Table 1:** ADMET of monomers A, B, and C using Grubbs-type catalyst ^a^.

Entry	Catalyst	Solvent	Temp. (°C)	t (h)	Yield ^b^ (%)	M_n_^c^ (Da)	M_w_^c^ (Da)	Ð^c^
Monomer A								
1	G2	toluene	80	12	73	2001	2207	1.10
2	G2	toluene	100	12	82	1714	1802	1.05
3	G2	toluene ^d^	80	24	81	1614	1666	1.03
4	G2	DCB	100	12	83	2021	2250	1.06
5	G-H2	toluene	80	12	78	1672	1745	1.04
6	G-H2	toluene	100	12	80	1705	1788	1.05
7	G2	toluene/DCB	80	24	76	1867	2029	1.09
Monomer B								
8	G2	toluene	80	24	81	1714	1791	1.04
9	G-H2	toluene	80	24	83	1372	1377	1.00
10	G2	toluene/DCB	80	24	79	2160	2374	1.10
Monomer C								
11	G2	toluene	80	24	76	2752	3558	1.29
12	G3	toluene/DCB	60	24	79	3355	3724	1.11
13	G3	toluene/DCB	60	48	75	3360	4066	1.21

^a^ All reactions were finished under a vacuum; 1 mL solvent, and 1 mol % of catalyst were used. Degasification after 6 h (monomer A) or 12 h (monomers B and C); ^b^ isolated yield by precipitation using methanol; ^c^ GPC data in THF against a polystyrene standard; ^d^ 0.5 mL toluene was used.

**Table 2:** Extinction coefficients of oligomers and copolymer.

Entry	Substance	A	ε (Lmol^−1^cm^−1^)	Equation ^a^
1	Oligomer A	0.39	7800	ε = A/BC (1)
2	Oligomer B	0.388	7760	
3	Oligomer C	0.386	7720	
4	Copolymer 1	1.05	21000	

^a^ ε Molar extinction coefficient (Lmol^−1^cm^−1^), A = absorbance, B = the length of light (cm), C = the concentration of substance (mol/L). ^b^ B = 1 cm, C = 5 × 10^−5^ M.

**Table 3:** Electrochemical date of oligomers.

Compound	Oxidation		Reduction	
	I (μA)	E (V)	I (μA)	E (V)
Oligomer A	1.02	0.57	0.99	0.43
Oligomer B	0.55	0.55	0.53	0.48
Oligomer C	0.57	0.54	0.56	0.47

**Table 4:** Copolymerization of monomer A with monomer D.

Entry	M_A_/M_D_	Yield (%) ^b^	M_n_ (Da) ^c^	M_w_ (Da) ^c^	Ð ^c^	x ^d^	y ^d^
1	1:4	76	13453	23824	1.77	5	25
2	1:2	80	6602	10893	1.65	2	12
3	1:1	78	2610	3973	1.52	2	4
4	2:1	79	2436	2901	1.19	3	2
5	4:1	75	1896	2058	1.09	3	1

^a^ All reactions were finished under a vacuum; 1 mL solvent and 1 mol % catalyst was used; ^b^ isolated yield by precipitation using methanol; ^c^ GPC data in THF against a polystyrene standard; ^d^ determined by ^1^H NMR and GPC; x = number of ferrocene units, y = number of fluorene units.

**Table 5:** Oxidation–reduction potential of copolymer 1 with different molecular weights.

Entry	x ^a^	y ^b^	E (V)
1	2	4	0.627
2	2	12	0.577
3	3	1	0.663
4	3	2	0.661

^a^ x = number of ferrocene units; ^b^ y = number of fluorene units.

## Supplementary Materials

The following are available online at https://www.mdpi.com/2073-4360/11/8/1334/s1, Figures S1–S11: ^1^H, ^13^C NMR spectra, and HRMS spectra of monomers and oligomer A, Figures S12–S19: GPC data, Figures S20–S25: ^1^H, ^13^C NMR spectra of oligomer A-C, Figures S26-S28: spectral analysis, Figures S29–S32: ^1^H, ^13^C NMR spectra of copolymers, Figures S33–S37: The color variance and cyclic voltammogram spectra.
