# Supplementary material for: Ferrocene-Containing Conjugated Oligomers Synthesized by Acyclic Diene Metathesis Polymerization
Source: Polymers (Basel). 2019 Aug 12;11(8):1334. doi: 10.3390/polym11081334 (PMC6722986; doi:10.3390/polym11081334)
Supplement: Supplementary file 1 [file polymers-11-01334-s001.pdf]

Supplementary Materials

Ferrocene-Containing Conjugated Oligomers Synthesized by Acyclic Diene Metathesis Polymerization

Xin Gao[1], Lei Deng[1], Jianfeng Hu[1,2], Hao Zhang[1,2,*]

[1]College of Chemical & Chemical Engineering, Inner Mongolia University, Hohhot 010021, P. R. China

[2]Inner Mongolia Key Laboratory of Fine Organic Synthesis, Hohhot 010021, P. R. China

Correspondence to: Hao Zhang (E-mail: *haozhang@imu.edu.cn; zh_hjf@hotmail.com*)

Table of Contents

1. ^{1}H, ^{13}C NMR spectra and HRMS spectra of monomers and oligomer A — S2

2. GPC data — S7

3. ^{1}H, ^{13}C NMR spectra of oligomer A - C — S11

4. Spectral analysis — S14

5. ^{1}H, ^{13}C NMR spectra of copolymers — S16

6. The colour variance and cyclic voltammogram spectra — S18

1. ^{1}H, ^{13}C NMR spectra and HRMS spectra of monomers and oligomer A

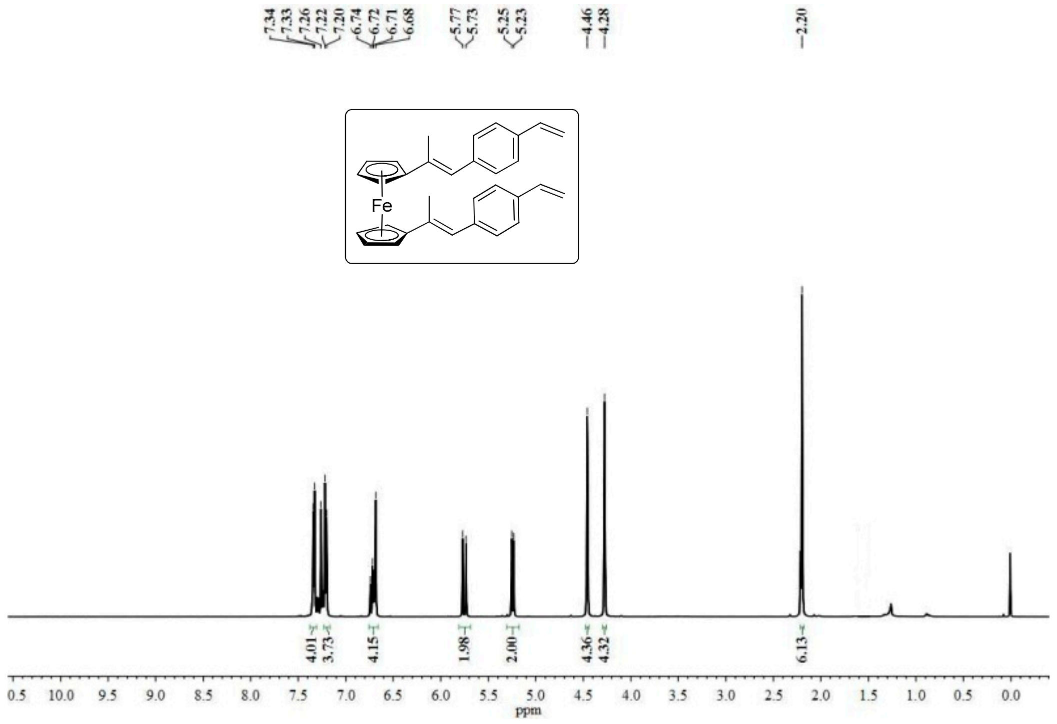

Figure S1 ^{1}H NMR (500 MHz) spectrum of monomer A measured in $CDCl_3$ at 25 °C.

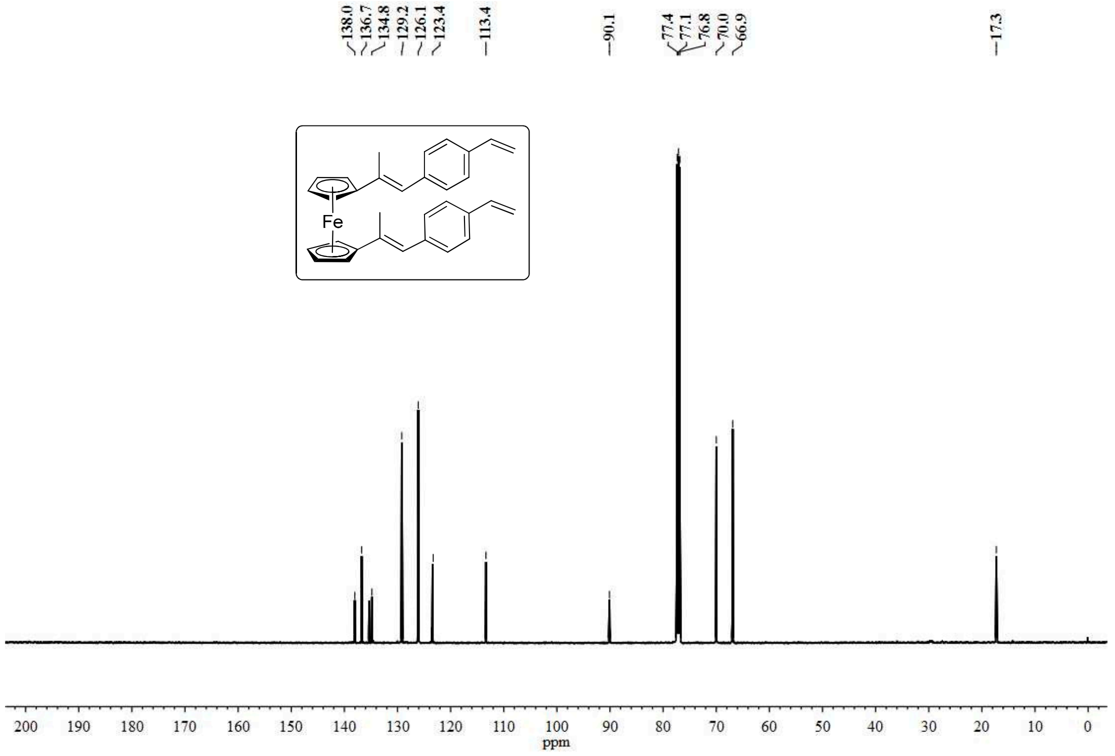

Figure S2 ^{13}C NMR (126 MHz) spectrum of monomer A measured in $CDCl_3$ at 25 °C.

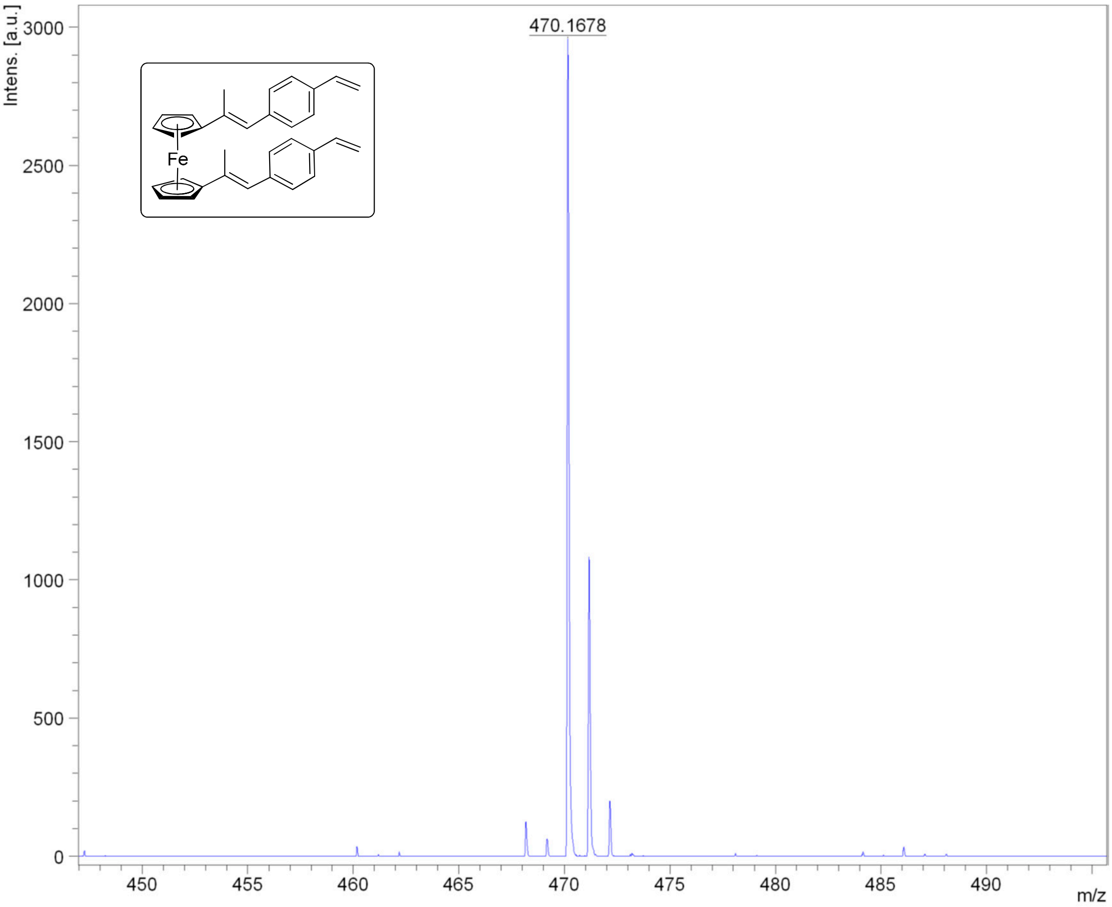

Figure S3 The HRMS (ESI-TOF) spectra of monomer A.

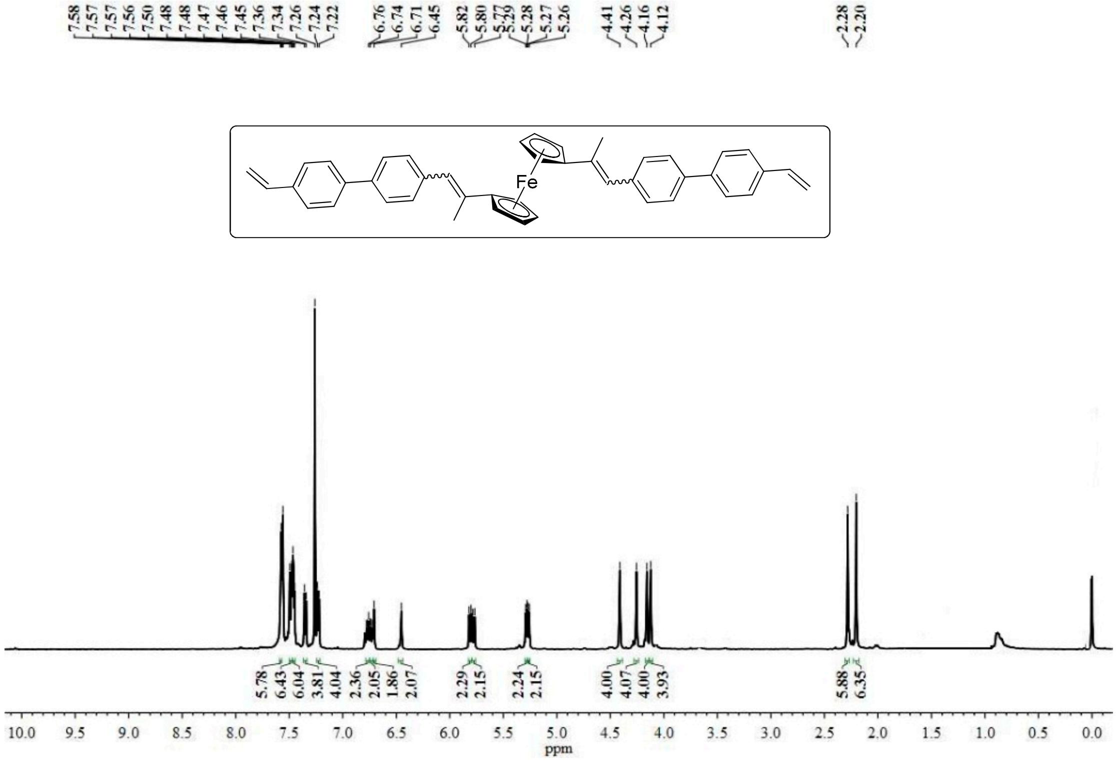

Figure S4 ^{1}H NMR (500 MHz) spectrum of monomer B measured in $CDCl_3$ at 25 °C.

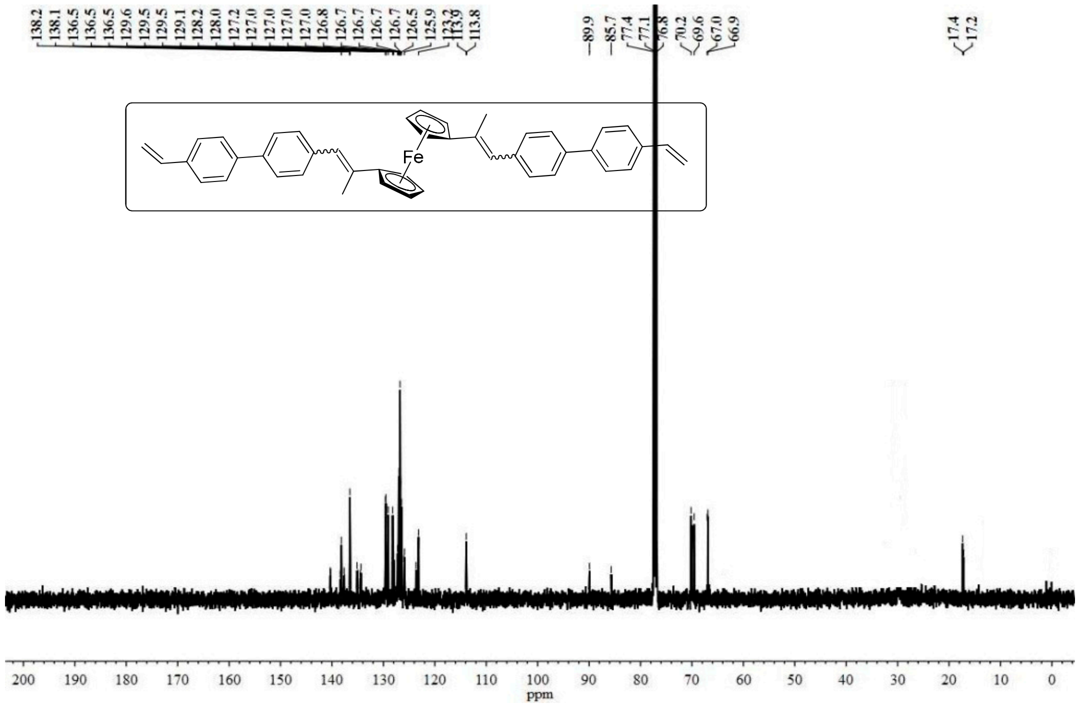

Figure S5 ^{13}C NMR (126 MHz) spectrum of monomer B measured in $CDCl_3$ at 25 °C.

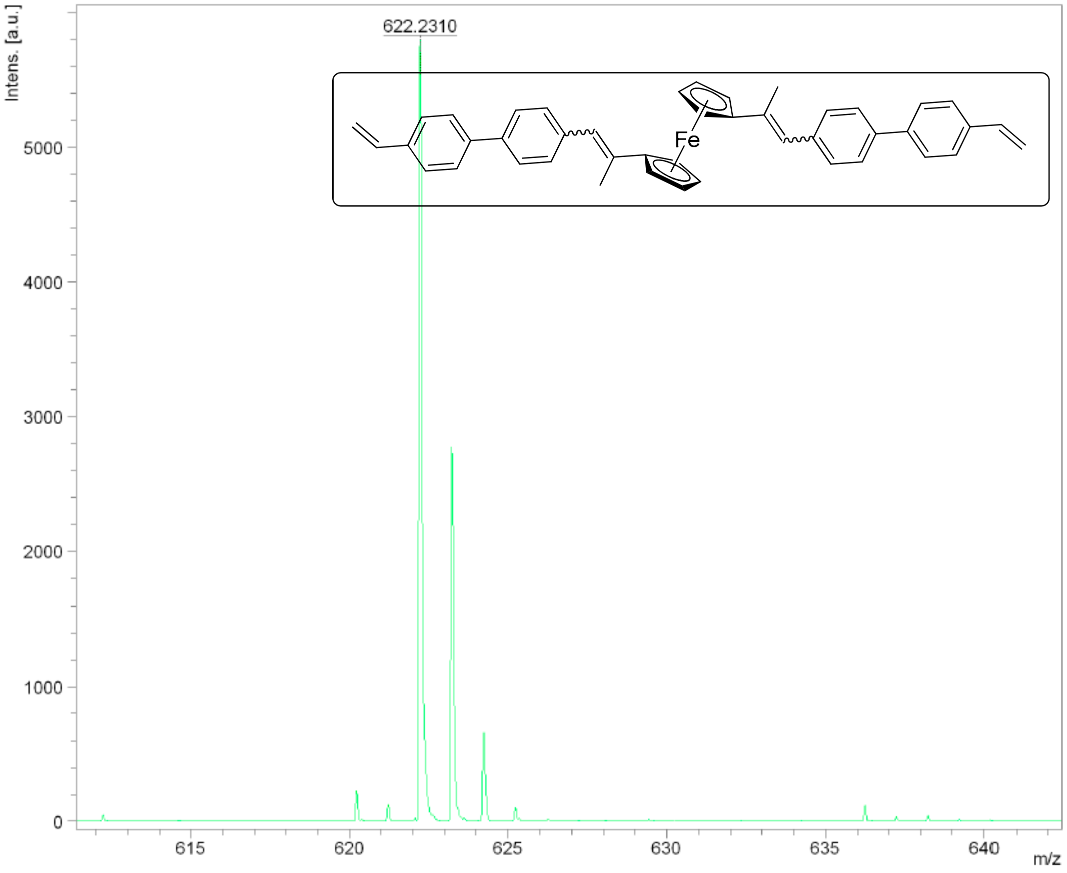

Figure S6 The HRMS (ESI-TOF) spectra of monomer B.

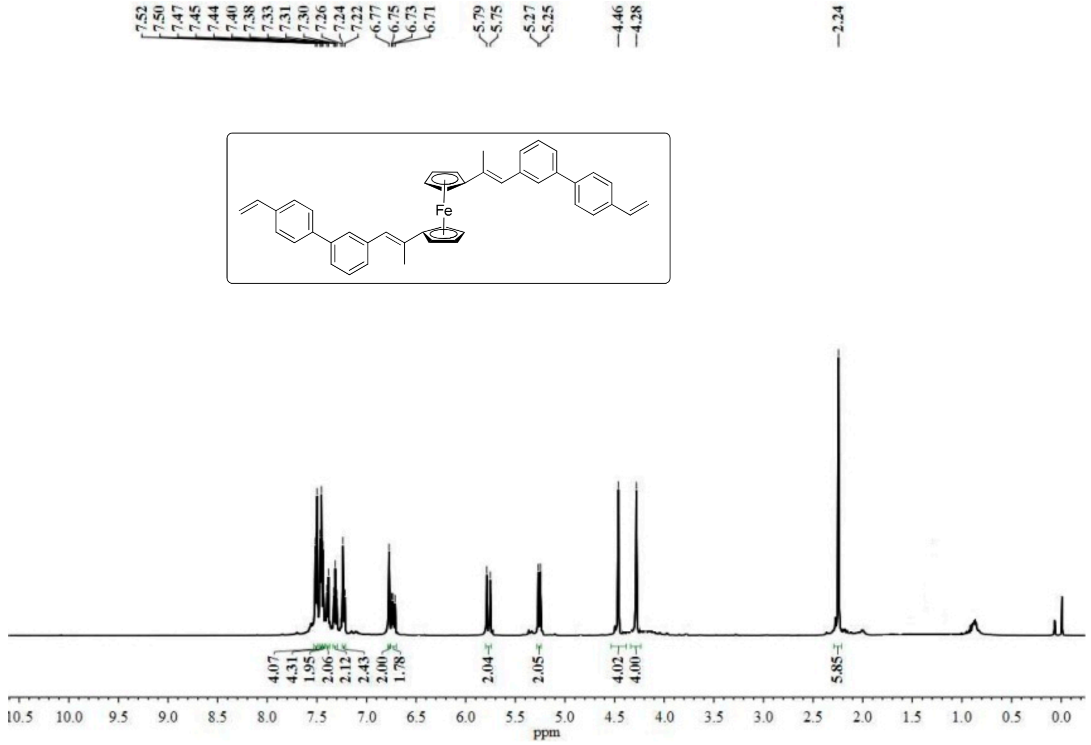

Figure S7 ^{1}H NMR (500 MHz) spectrum of monomer C measured in $CDCl_3$ at 25 °C.

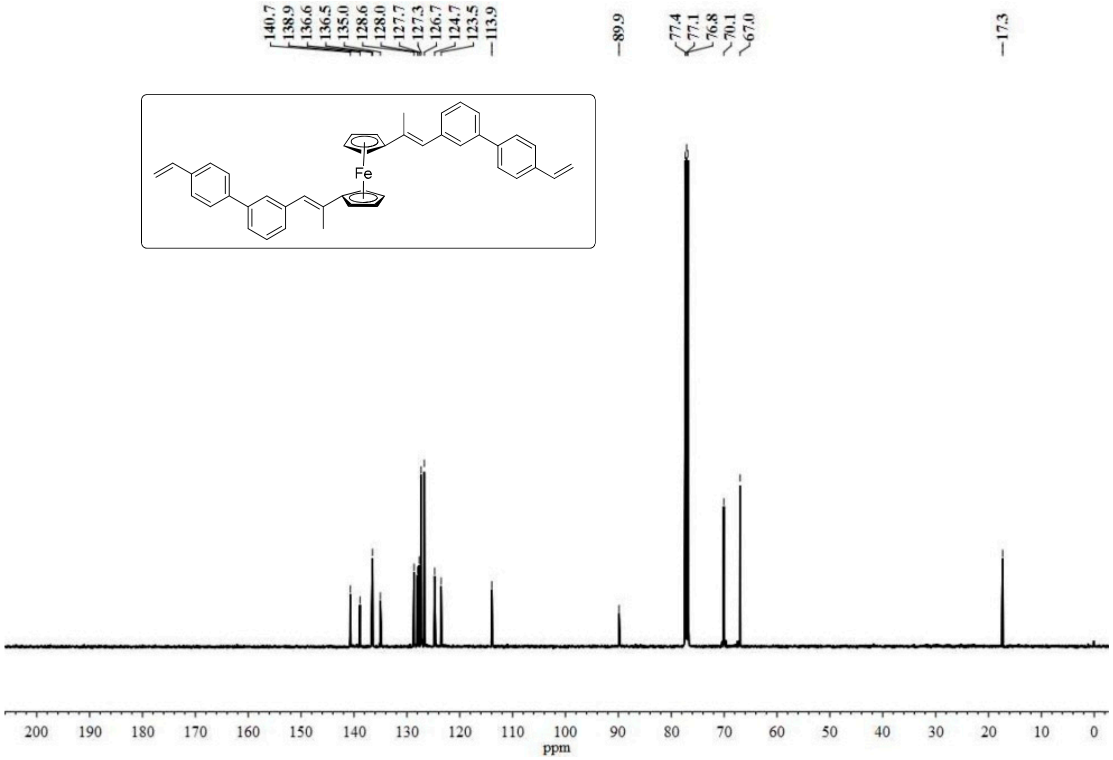

Figure S8 ^{13}C NMR (126 MHz) spectrum of monomer C measured in $CDCl_3$ at 25 °C.

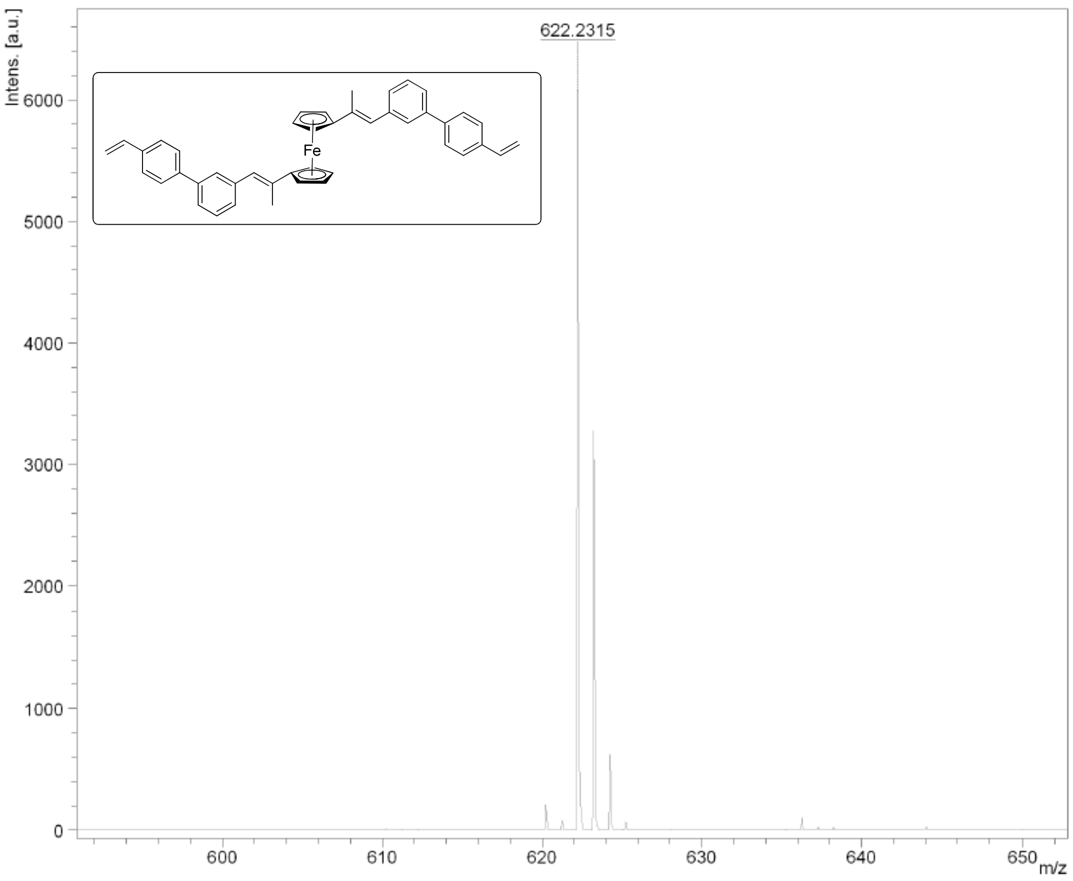

Figure S9 The HRMS (ESI-TOF) spectra of monomer C.

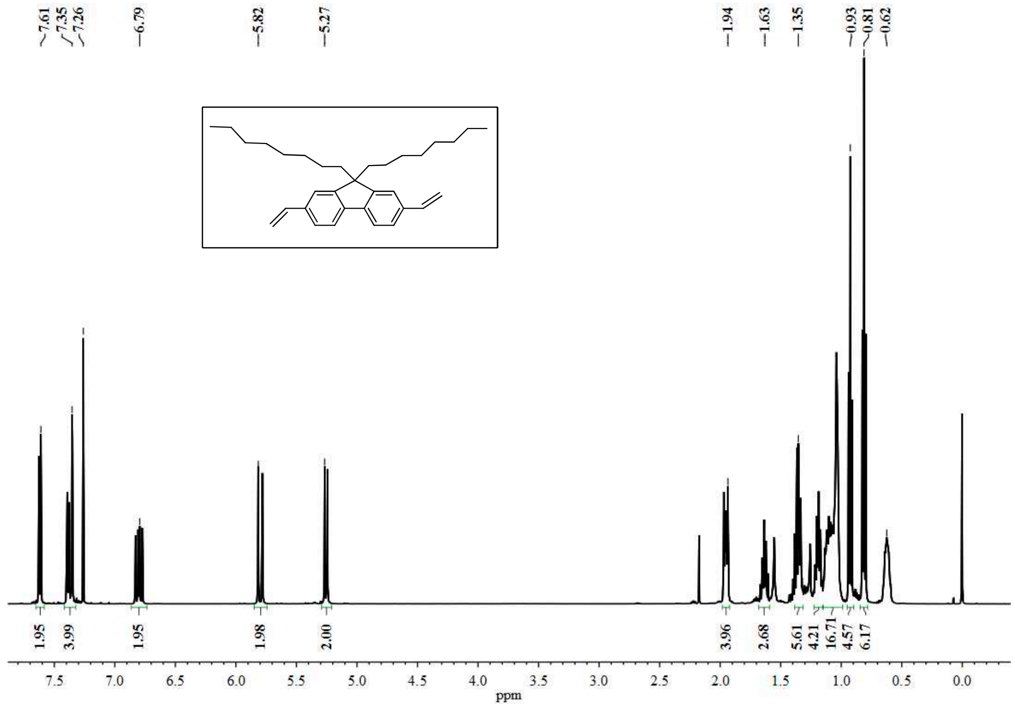

Figure S10 ^{1}H NMR (500 MHz) spectrum of monomer D measured in $CDCl_3$ at 25 °C.

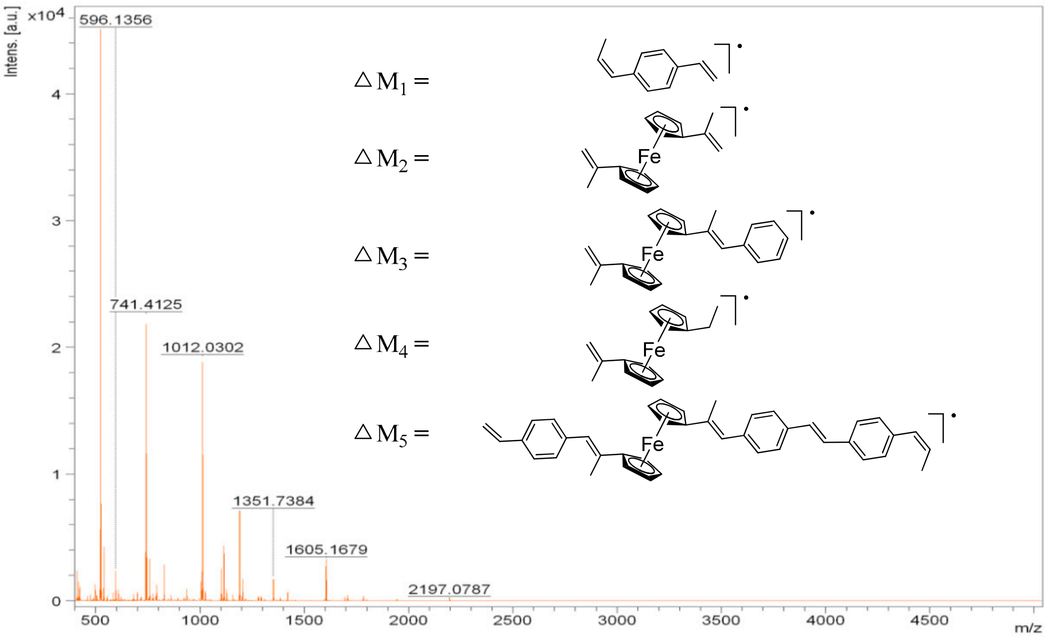

Figure S11 MALDI-TOF spectrum of oligomer A (M_n = 2021, PDI = 1.06, Table 1, entry 4)

2. GPC data

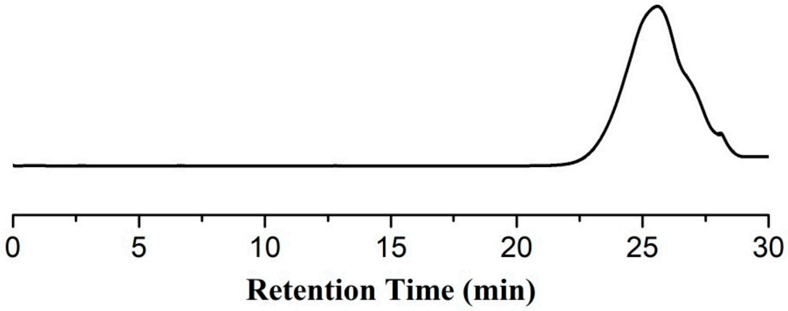

Figure S12 GPC data of oligomer A (Table 1, entry 1)

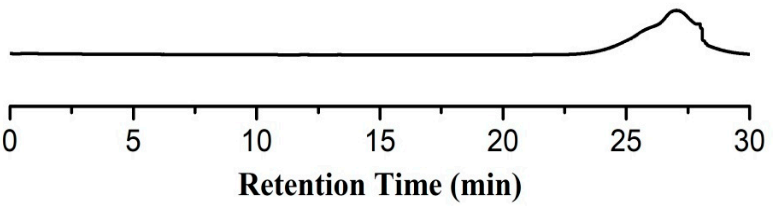

Figure S13 GPC data of oligomer A (Table 1, entry 3)

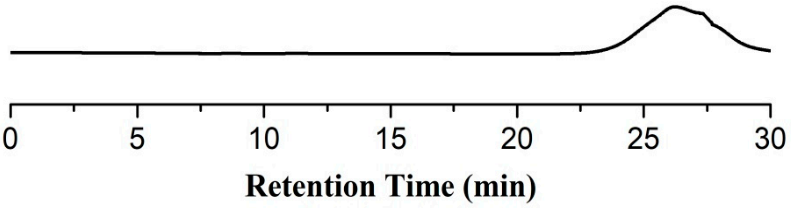

Figure S14 GPC data of oligomer B (Table 1, entry 8)

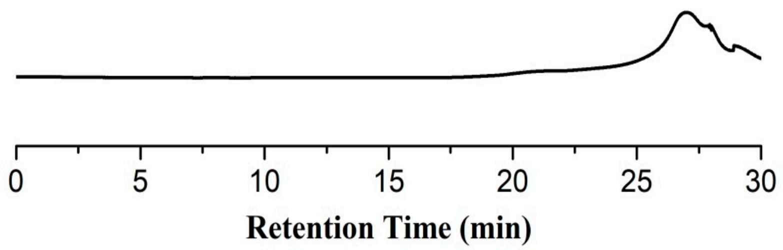

Figure S15 GPC data of oligomer B (Table 1, entry 9)

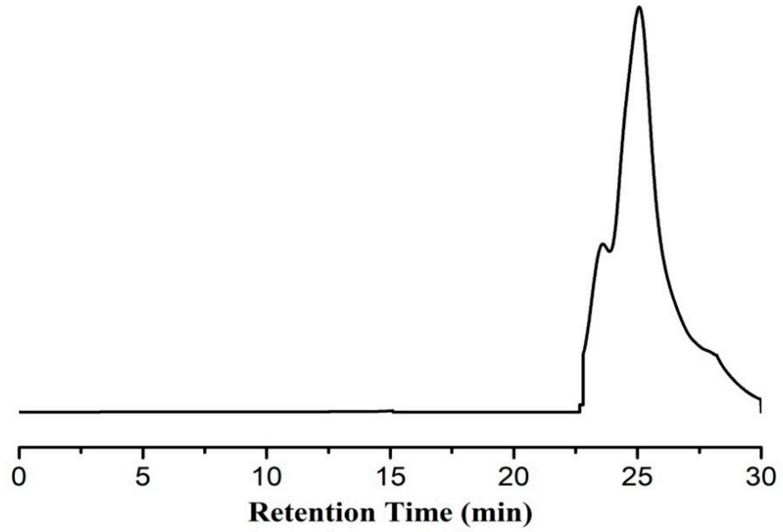

Figure S16 GPC data of oligomer B (Table 1, entry 10)

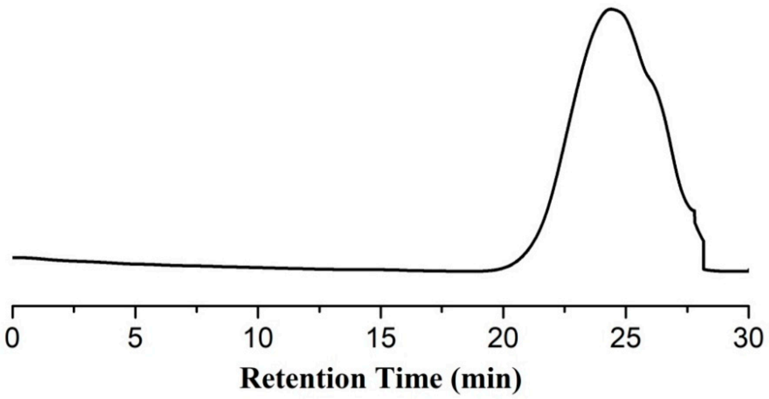

Figure S17 GPC data of oligomer C (Table 1, entry 11)

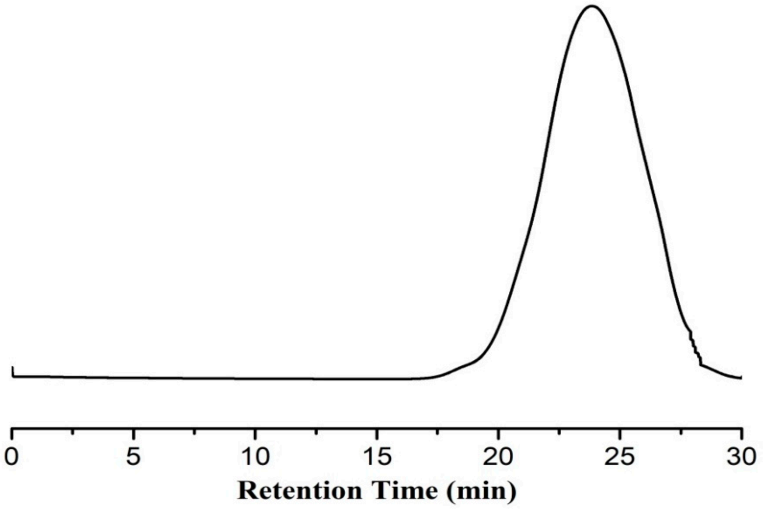

Figure S18 GPC data of oligomer C (Table 1, entry 13)

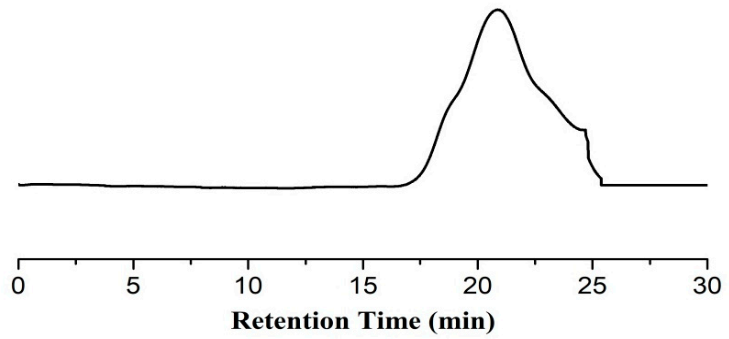

Figure S19 GPC data of copolymer 1 (Table 3, entry 1)

3. ^{1}H, ^{13}C NMR spectra of oligomer A - C

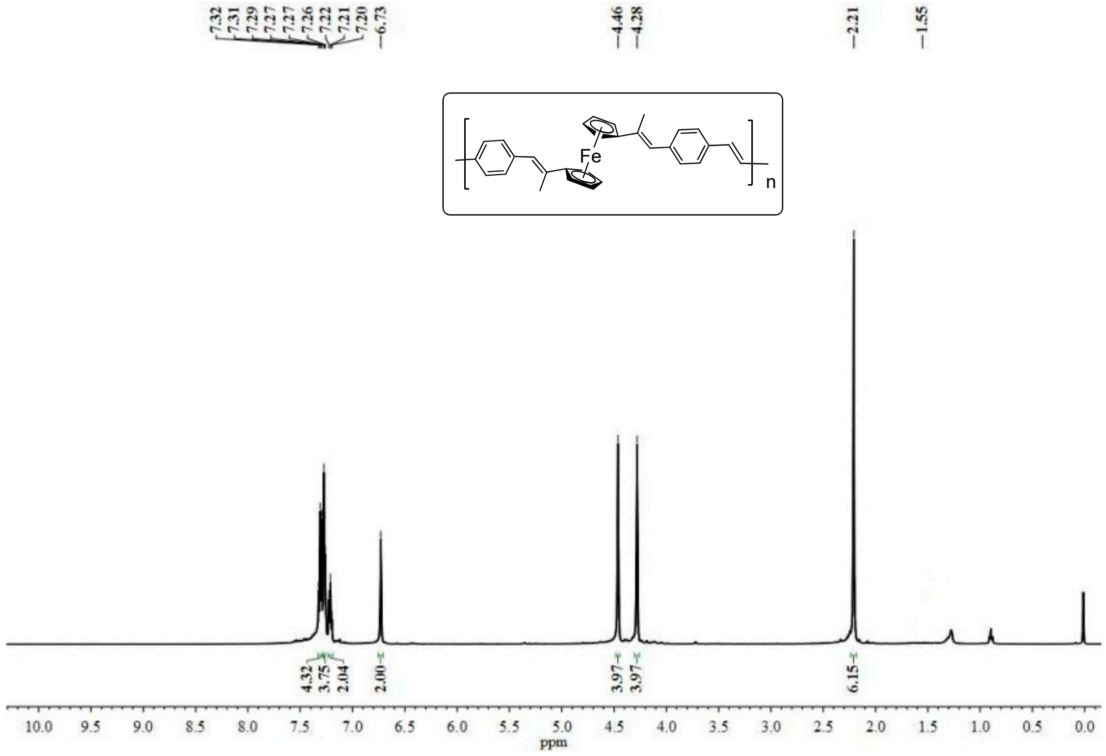

Figure S20 ^{1}H NMR (500 MHz) spectrum of oligomer A measured in $CDCl_3$ at 25 °C.

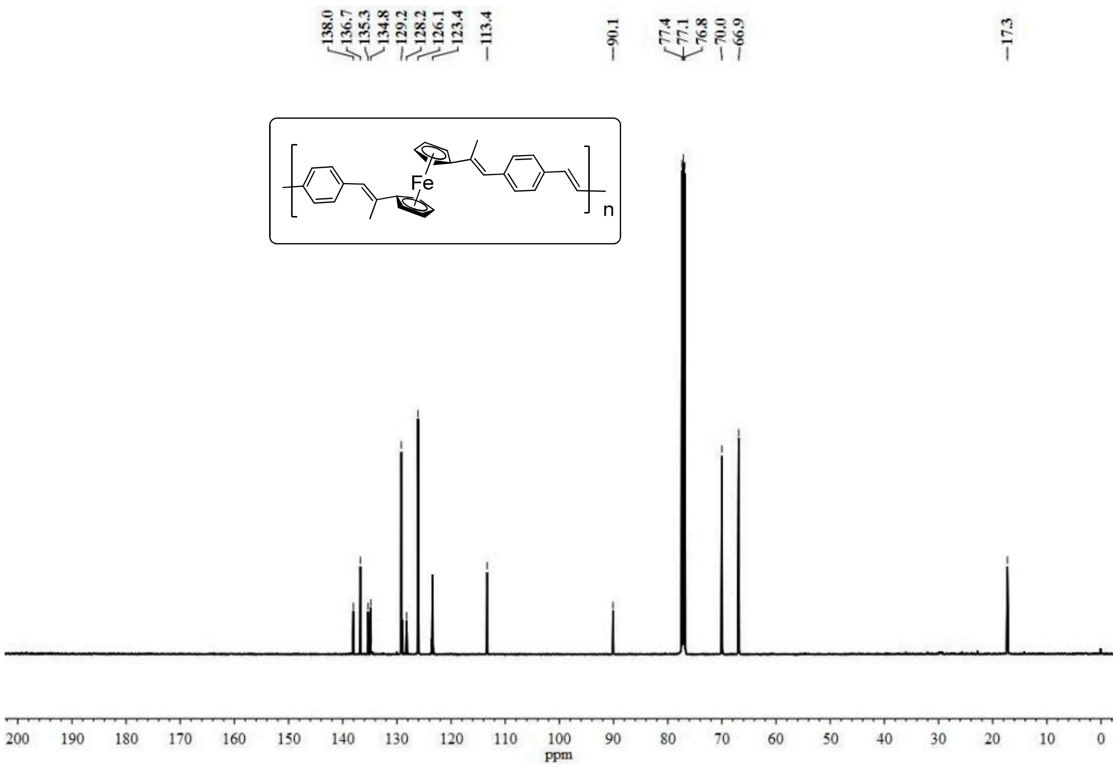

Figure S21 ^{13}C NMR (126 MHz) spectrum of oligomer A measured in $CDCl_3$ at 25 °C.

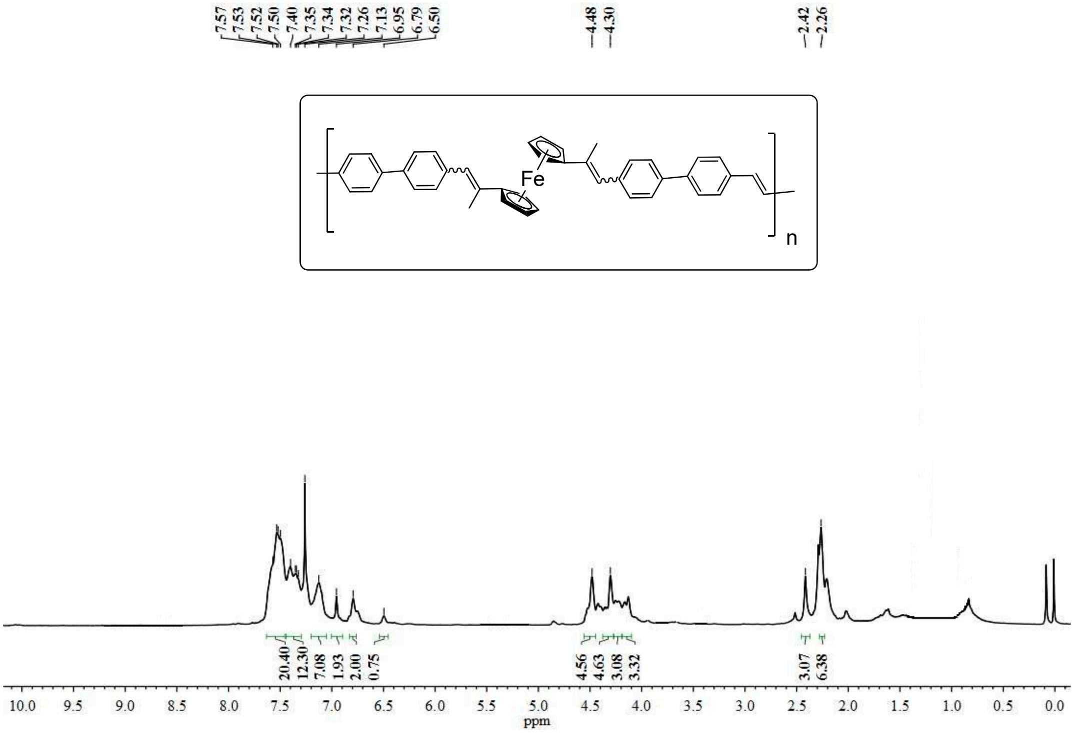

Figure S22 ^{1}H NMR (500 MHz) spectrum of oligomer B measured in $CDCl_3$ at 25 °C.

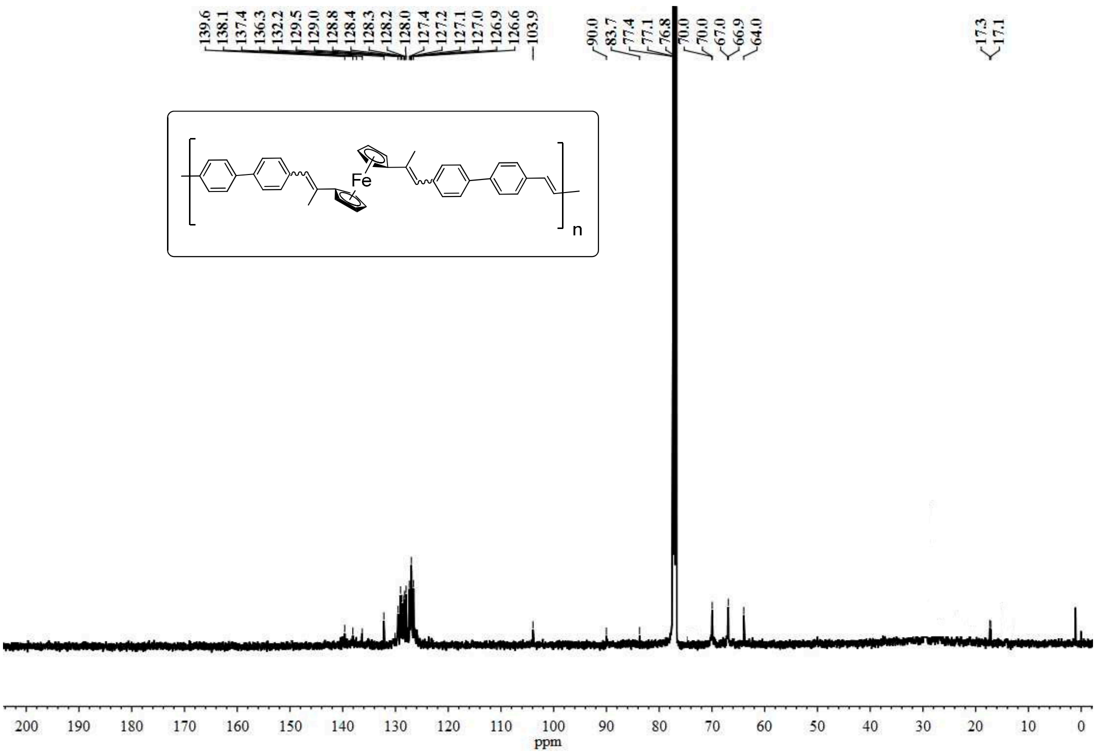

Figure S23 ^{13}C NMR (126 MHz) spectrum of oligomer B measured in $CDCl_3$ at 25 °C.

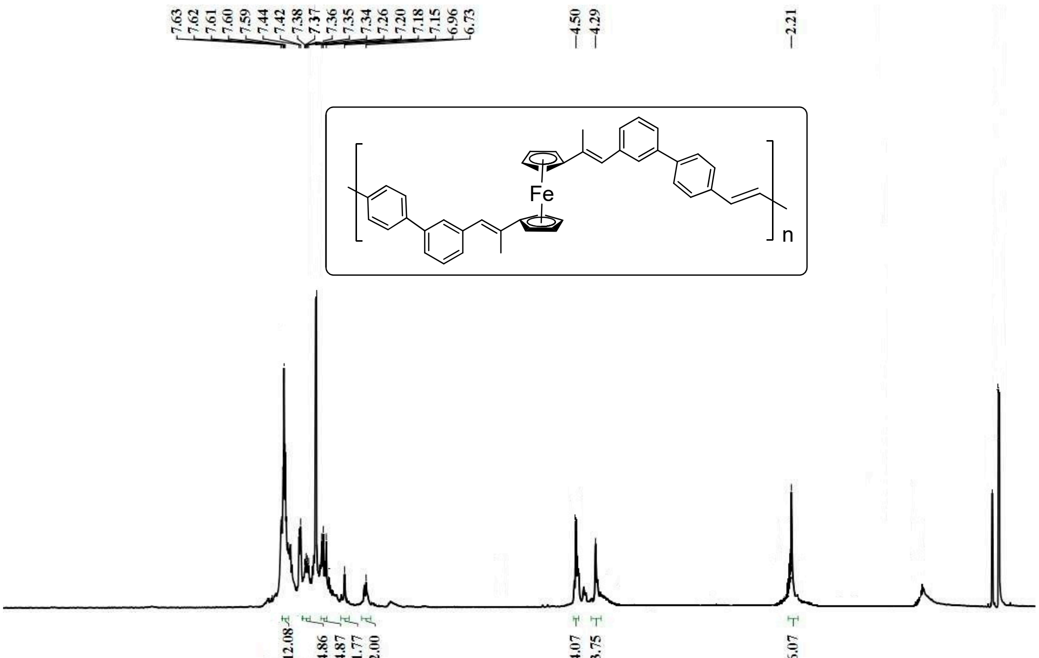

Figure S24 ^{1}H NMR (500 MHz) spectrum of oligomer C measured in $CDCl_3$ at 25 °C.

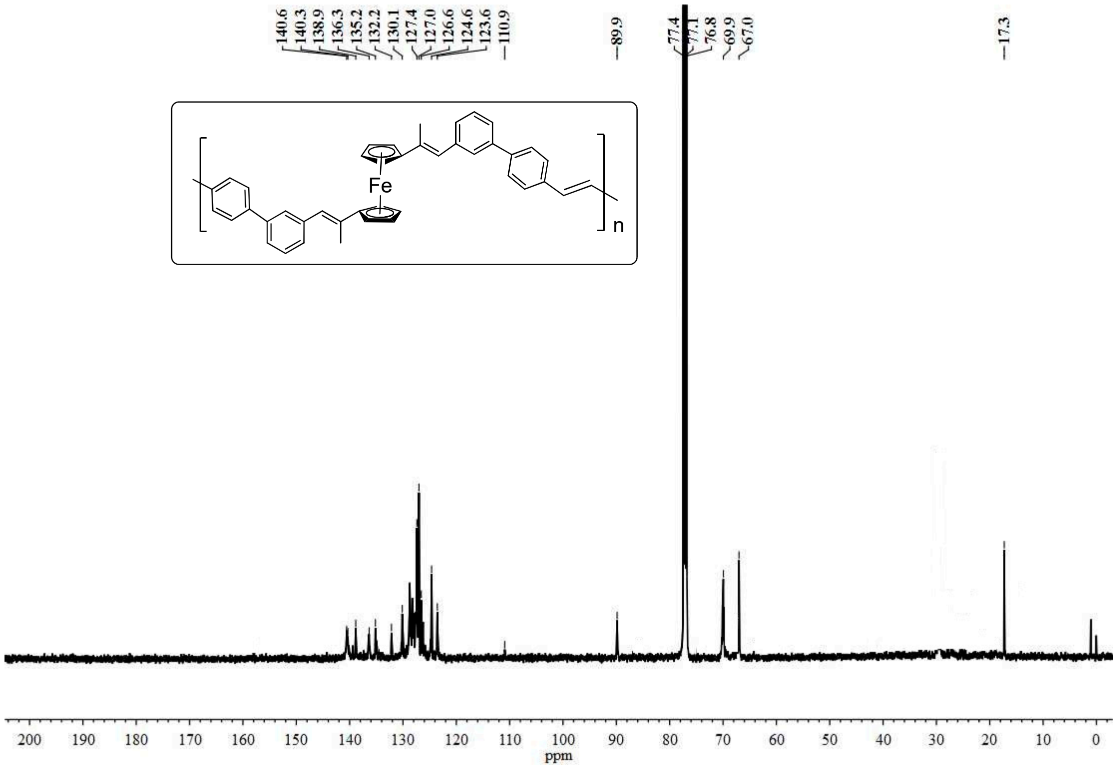

Figure S25 ^{13}C NMR (126 MHz) spectrum of oligomer C measured in $CDCl_3$ at 25 °C.

4. Spectral analysis

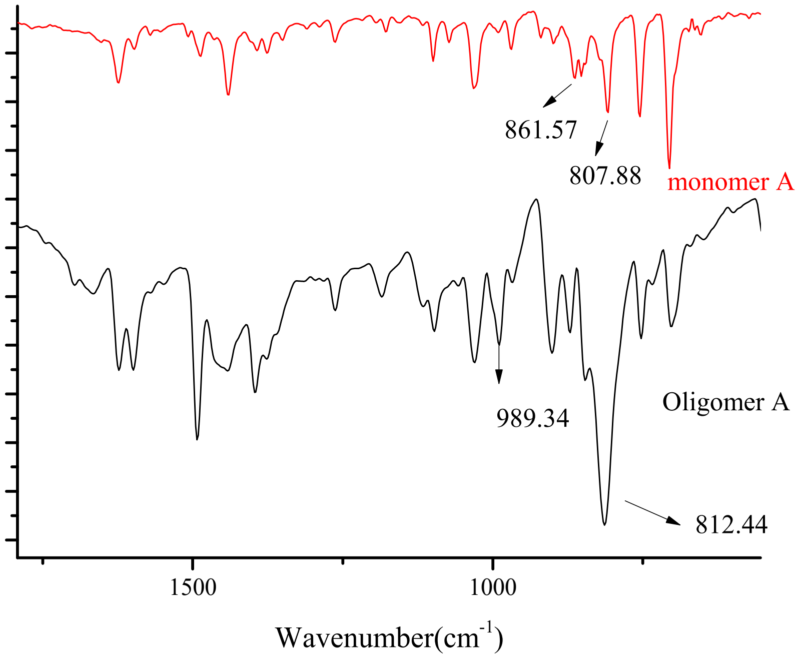

Figure S26 FT-IR spectra (selected region) of monomer A and its ADMET product

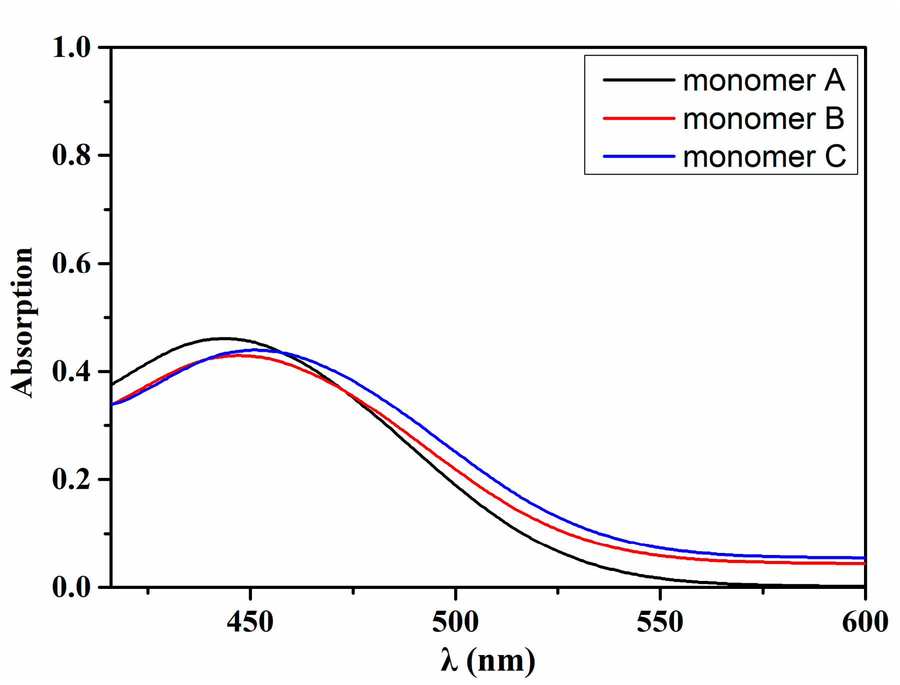

Figure S27 UV-vis spectra of monomer A, B and C in CH_2Cl_2.

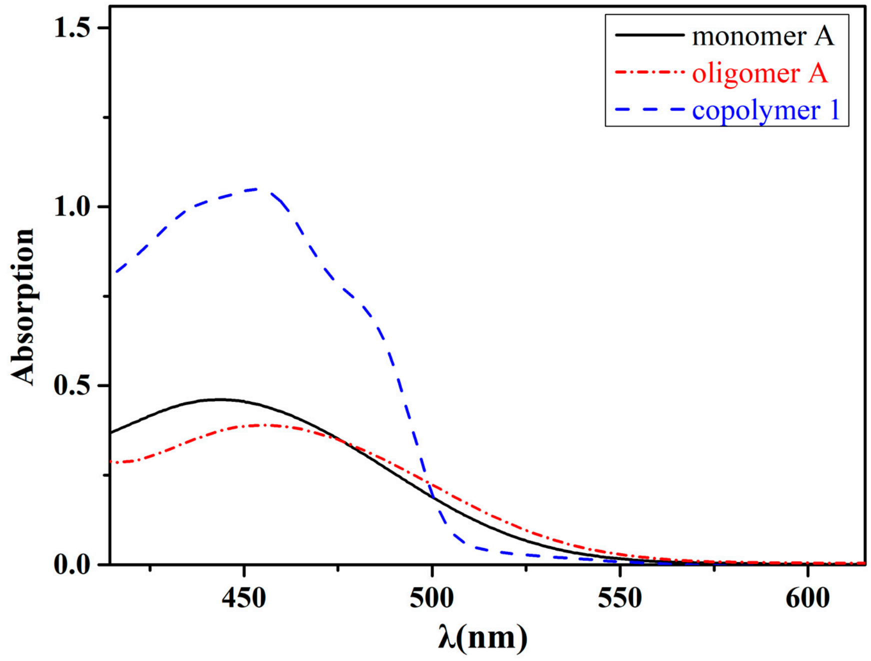

Figure S28 UV-vis spectra of monomer A, oligomer A and copolymer 1.

5. ^{1}H, ^{13}C NMR spectra of copolymers

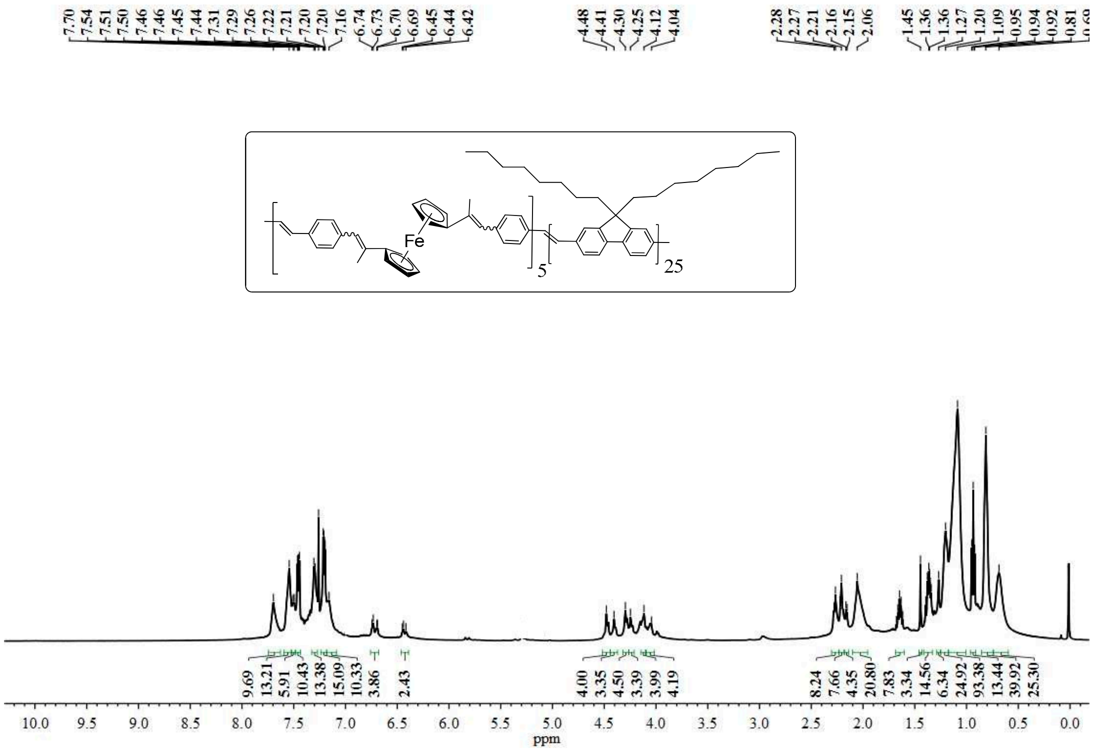

Figure S29 ^{1}H NMR spectra of copolymer 1 (n_A **:** n_{flu} = 1:4) (Table 3, entry 1)

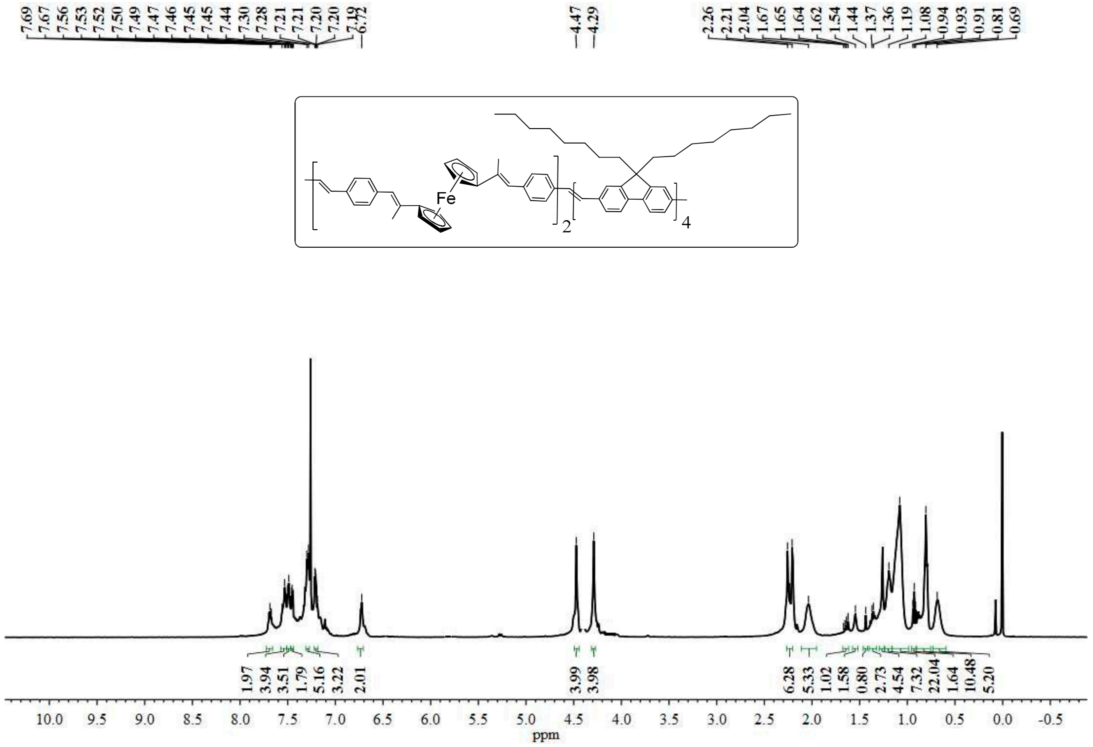

Figure S30 ^{1}H NMR spectra of copolymer 1 (n_A **:** n_{flu} = 1:1) (Table 3, entry 3)

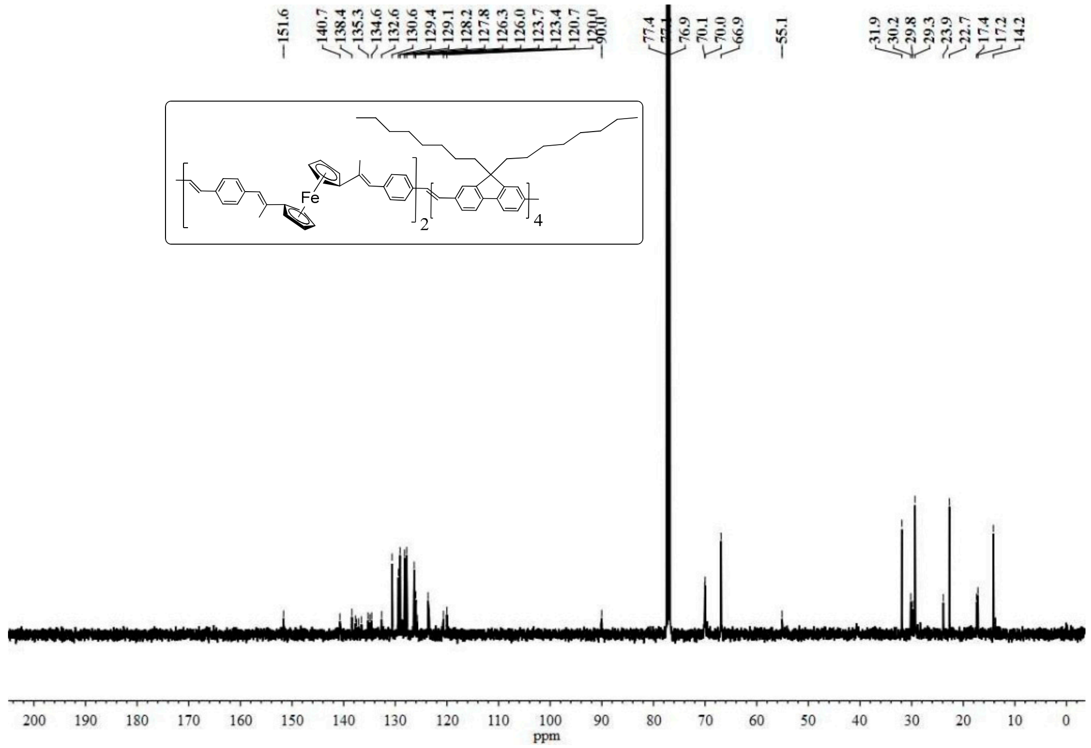

Figure S31 ^{13}C NMR spectra of copolymer 1 ($n_A : n_{flu} = 1:1$) (Table 3, entry 3)

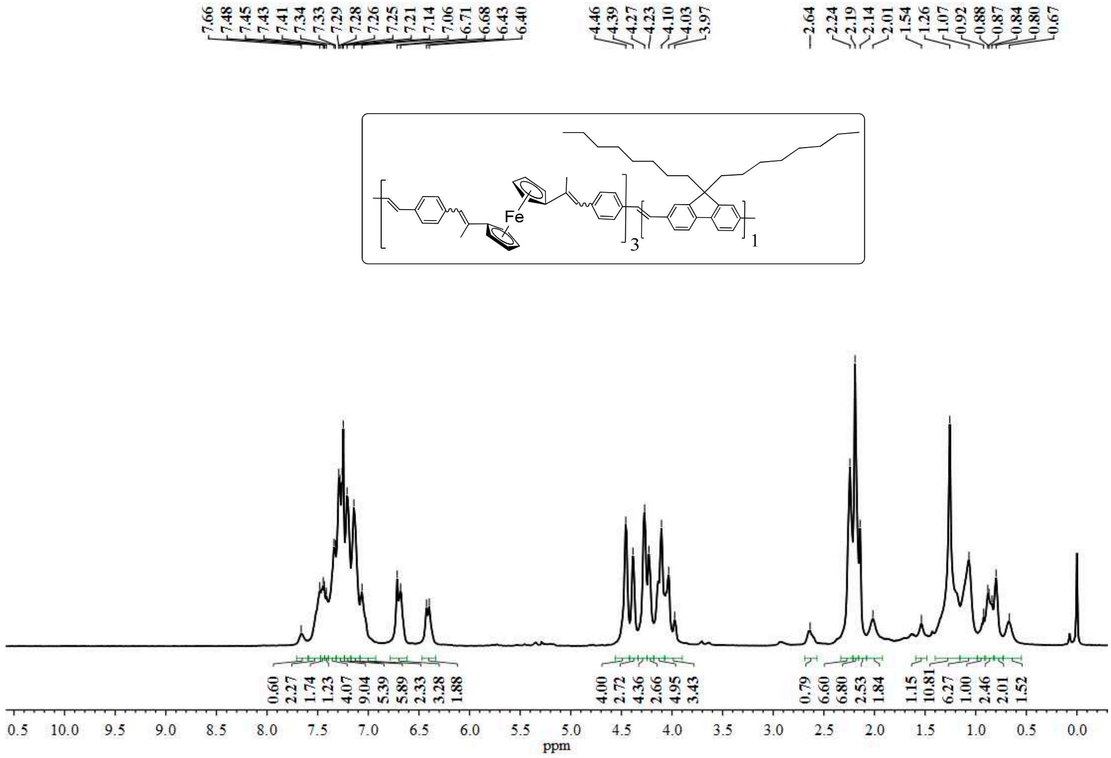

Figure S32 ^{1}H NMR spectra of copolymer 1 ($n_A : n_{flu} = 4:1$) (Table 3, entry 5)

6. The color variance and cyclic voltammogram spectra

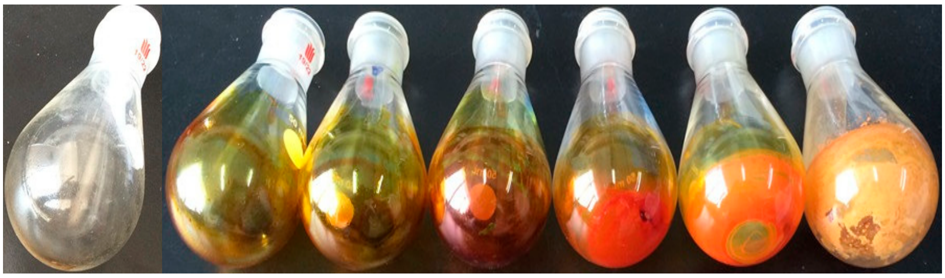

Figure S33 The color variance of polymer D (left), copolymer 1 and oligomer A (right)

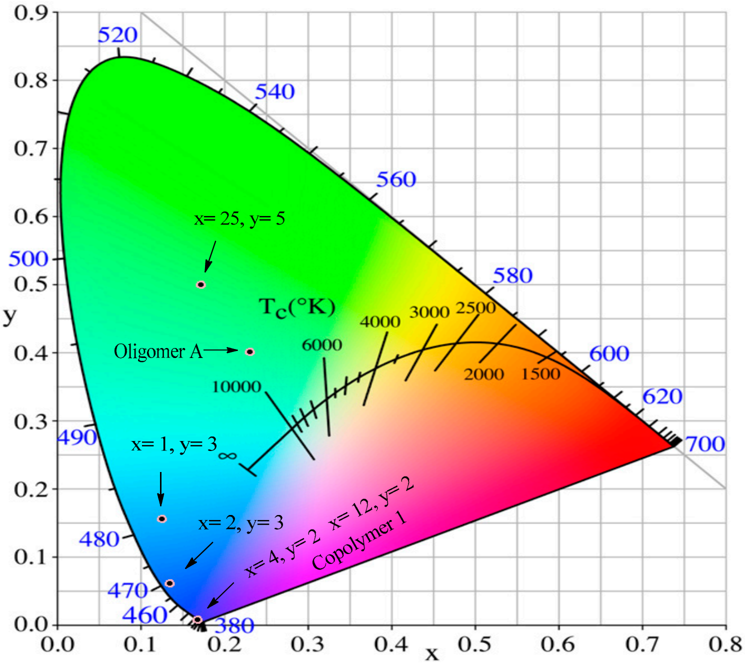

Figure S34 The CIE plot of copolymer 1 with different molecular weight and oligomer A

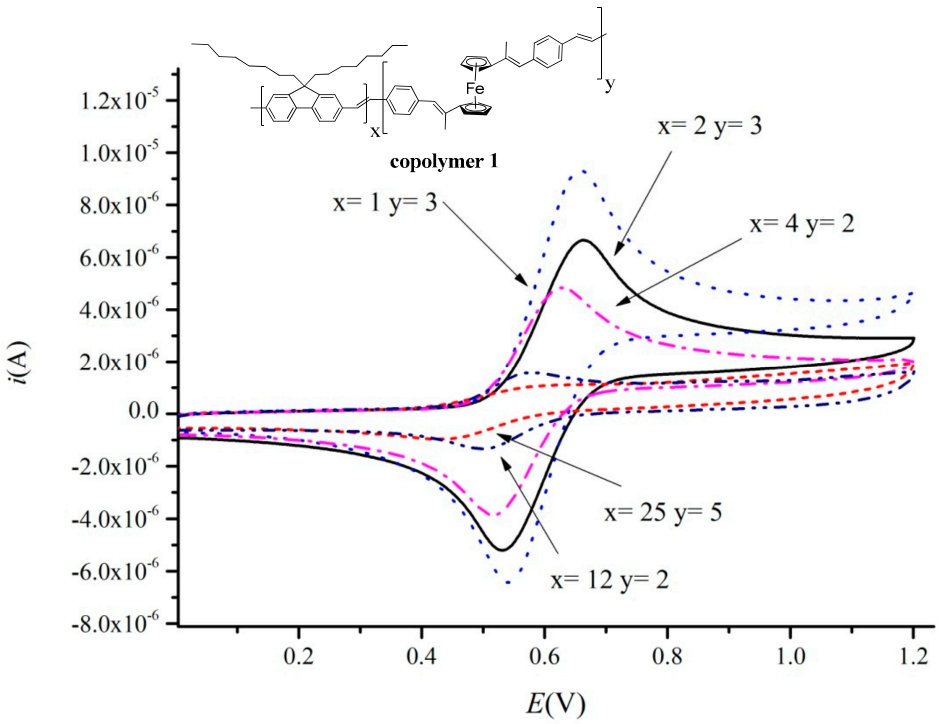

Figure S35 Cyclic voltammogram spectra of copolymer 1 with different molecular weight

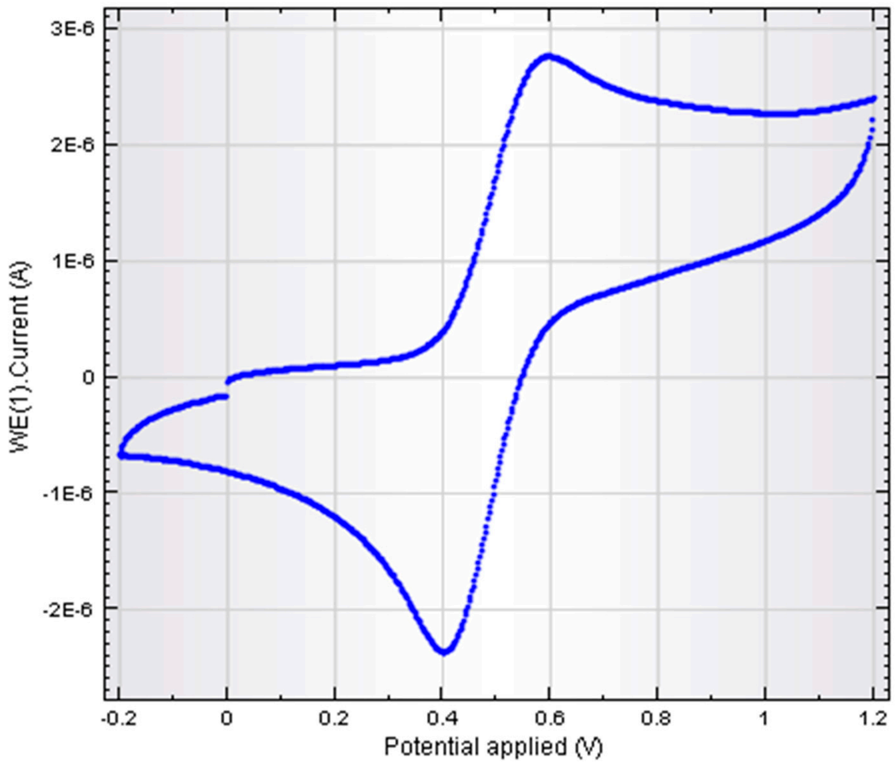

Figure S36 Cyclic voltammogram spectra of monomer B

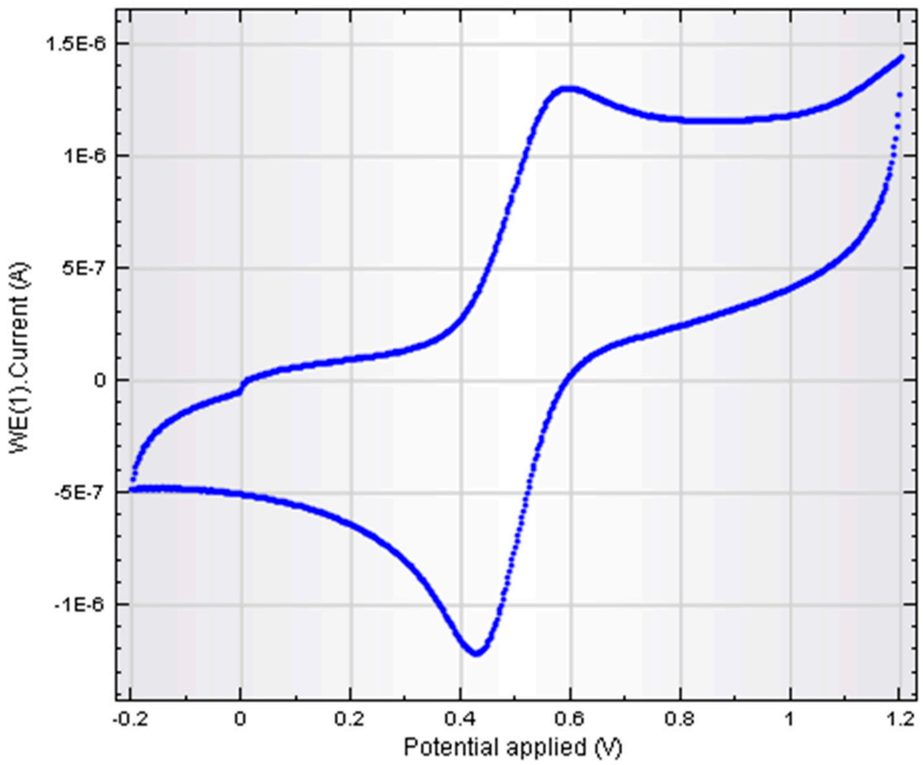

Figure S37 Cyclic voltammogram spectra of monomer C
